# Comparative Proteomic Analysis of Liver Tissues and Serum in *db*/*db* Mice

**DOI:** 10.3390/ijms23179687

**Published:** 2022-08-26

**Authors:** Yu Zhang, Xiumei Wu, Mengyun Xu, Tong Yue, Ping Ling, Tingyu Fang, Sihui Luo, Suowen Xu, Jianping Weng

**Affiliations:** 1 Department of Endocrinology, Institute of Endocrine and Metabolic Diseases, The First Affiliated Hospital of USTC, Division of Life Sciences and Medicine, Clinical Research Hospital of Chinese Academy of Sciences (Hefei), University of Science and Technology of China, Hefei 230001, China; 2 Department of Endocrinology and Metabolic Disease, The Third Affiliated Hospital of Sun Yat-sen University, Guangzhou 510000, China

**Keywords:** biomarkers, differentially expressed proteins, diabetes, NAFLD, TMT-labeling proteomic analysis

## Abstract

Background and Aims: Non-alcoholic fatty liver disease (NAFLD) affects one-quarter of individuals worldwide. Liver biopsy, as the current reliable method for NAFLD evaluation, causes low patient acceptance because of the nature of invasive sampling. Therefore, sensitive non-invasive serum biomarkers are urgently needed. Results: The serum gene ontology (GO) classification and Kyoto encyclopedia of genes and genomes (KEGG) analysis revealed the DEPs enriched in pathways including JAK-STAT and FoxO. GO analysis indicated that serum DEPs were mainly involved in the cellular process, metabolic process, response to stimulus, and biological regulation. Hepatic proteomic KEGG analysis revealed the DEPs were mainly enriched in the PPAR signaling pathway, retinol metabolism, glycine, serine, and threonine metabolism, fatty acid elongation, biosynthesis of unsaturated fatty acids, glutathione metabolism, and steroid hormone biosynthesis. GO analysis revealed that DEPs predominantly participated in cellular, biological regulation, multicellular organismal, localization, signaling, multi-organism, and immune system processes. Protein-protein interaction (PPI) implied diverse clusters of the DEPs. Besides, the paralleled changes of the common upregulated and downregulated DEPs existed in both the liver and serum were validated in the mRNA expression of NRP1, MUP3, SERPINA1E, ALPL, and ALDOB as observed in our proteomic screening. Methods: We conducted hepatic and serum proteomic analysis based on the leptin-receptor-deficient mouse (*db*/*db*), a well-established diabetic mouse model with overt obesity and NAFLD. The results show differentially expressed proteins (DEPs) in hepatic and serum proteomic analysis. A parallel reaction monitor (PRM) confirmed the authenticity of the selected DEPs. Conclusion: These results are supposed to offer sensitive non-invasive serum biomarkers for diabetes and NAFLD.

## 1. Introduction

Affecting a quarter of the worldwide population, non-alcoholic fatty liver disease (NAFLD) contributes detrimental risks to a series of metabolic diseases such as type 2 diabetes, obesity, dyslipidemia, and cardiovascular diseases. Besides, unresolved NAFLD could progressively advance to non-alcoholic steatohepatitis (NASH), liver fibrosis, cirrhosis, and even hepatocellular carcinoma (HCC), which poses serious challenges to world public health [1,2,3,4]. No pharmaceuticals have been approved by the Food and Drug Administration (FDA) to treat NAFLD except for losing weight through dieting and exercise. With effective treatments, the progression of NAFLD is reversible between the initial pathophysiologic stages of non-alcoholic fatty liver (NAFL) and NASH, and thus therapeutic targets for these two stages are essential to slow down NAFLD progression and improve prognosis [5]. Effective clinical diagnosis for the early stages of NAFLD is the keystone for timely treatment. Currently, the available biomarkers encompass imaging biomarkers and blood biomarkers, and panels for the early stage of NAFLD. Imaging biomarkers include abdominal ultrasonography, controlled attenuation parameter, and MRI—estimated proton density fat fraction. The blood biomarkers and panels are listed as follows: fatty liver index: body mass index, waist circumference, triglycerides, and gamma-glutamyltransferase (GGT); hepatic steatosis index: aspartate aminotransferase (AST): alanine aminotransferase (ALT) ratio, BMI, female sex, and diabetes mellitus; NAFLD liver fat score: metabolic syndrome, type 2 diabetes mellitus, fasting serum insulin, fasting serum AST and AST: ALT ratio; SteatoTest: six components of the Fibro Test- Acti Test plus BMI, cholesterol, triglycerides, and glucose adjusted for age and sex; NAFLD ridge score: ALT, HDL cholesterol, triglycerides, hemoglobin A1c (HbA1c), white blood cell count, and hypertension [6]. Although diverse NAFLD diagnosis methods such as MRI or FibroScan have been explored and entered into clinical uses, the limitations of the misleading interpretation based on the visual image make liver biopsy as irreplaceable as the golden standard diagnosis technique [7]. However, the surgical complications brought by the invasive biopsy, such as peritoneal effusion, lowers patient acceptance, which increases barriers to accurate clinical diagnosis [4,8]. Besides, the aforementioned blood biomarkers and panels performed no better than imaging biomarkers in NAFLD diagnosis [6]. Herein, there is an urgent need to explore novel sensitive non-invasive biomarkers for precisely judging the severity of NAFLD [9].

Proteins are the executors of all life activities, playing critical roles in cellular function [10]. Recently, TMT (tandem mass tag)-labeling proteomic analysis has been recommended as a dependable method for accurately quantifying relative protein levels in complex samples due to its technical strengths such as good sensitivity, reproducibility, and signal-to-noise ratio [11,12]. Ample studies have employed this method to discover potential biomarkers that are closely correlated to the phenotypes of diseases including NAFLD [10,13,14,15,16].

Leptin-receptor-deficient (*db*/*db*) mice are widely used as diabetic animal models accompanied by obesity and liver steatosis, automatically generating hyperglycemia with insulin resistance under standard feedings [17]. Therefore, the *db*/*db* mouse model recapitulates features of metabolic syndromes like obesity, hyperglycemia, and dyslipidemia observed among NAFLD patients [8,11]. Herein, this study aimed to explore potential non-invasive biomarkers related to the phenotype of multiple metabolic syndromes of NAFLD by performing TMT-labeling proteomic analysis in the liver and serum samples of *db*/*db* mice. Quantitative differentially expressed proteins (DEPs) were validated by utilizing a parallel-reacted monitor (PRM) analysis, and the overlapped proteins between the samples of liver tissues and serum were confirmed at the transcriptional level. Furthermore, the data of our proteomic analysis originating from the liver and serum of *db*/*db* mice could serve as resources for future studies related to NAFLD biomarkers.

## 2. Results

### 2.1. Validation of NAFLD Mouse Model Based on db/db Mice

**Figure 1:**
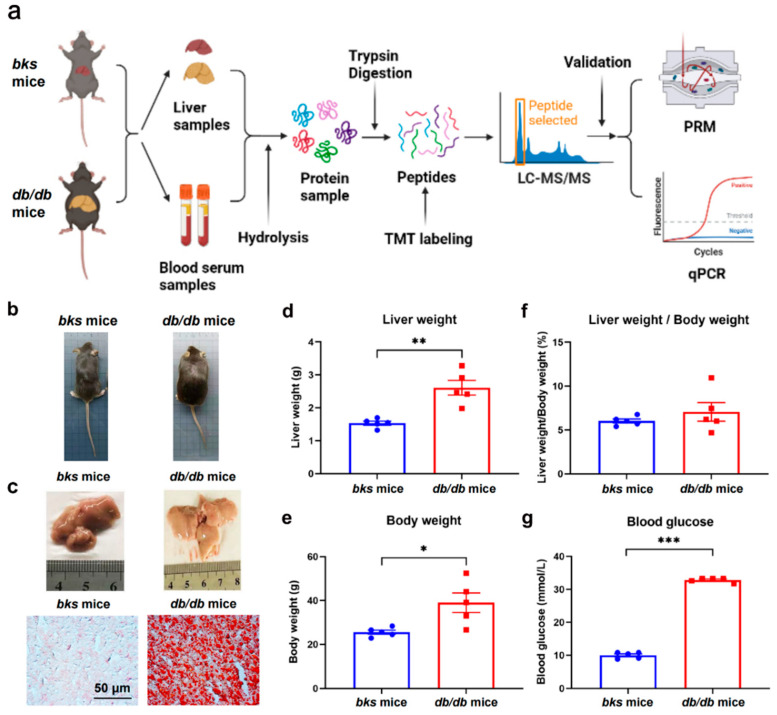
Study workflow and the validation of the genetic NAFLD mouse model. (**a**) Workflow chart; (**b**) Representative phages of *db*/*db* and *bks* mice (*n* = 5); (**c**) Representative liver phages and the corresponding Oil Red O staining of *bks* and *db*/*db* mice; (**d**) Liver weight, (**e**) body weight, (**f**) ratios of liver and body weight and (**g**) blood glucose of *db*/*db* mice and *bks* control group. Red dots represent *bks* mice and blue squares represent *db/db* mice. * *p* < 0.05; ** *p* < 0.01; *** *p* < 0.001.

As indicated in Figure 1a, mice were fed a chow diet for 12 weeks, then liver and serum samples were taken for TMT-labeling quantitative proteomic analysis. Subsequently, PRM and qPCR methods were utilized to validate the DEPs. After 12 weeks, *db*/*db* mice were heavier than the *bks* mice group (Figure 1b), and the *db*/*db* mice presented more severe liver steatosis as indicated by the results of liver phages and the Oil Red O staining (Figure 1c). Moreover, as shown in Figure S1 and Table S1, lipid-droplet-related markers such as fatty acid-binding protein (*Fabp4)*, perilipin-4 (*Plin4)*, perilipin-2 (*Plin2)*, perilipin-3 (*Plin3)*, perilipin-5 (*Plin5),* and ferroptosis suppressor protein 1 (*Aifm2)* were also upregulated in *db*/*db* mouse livers. In addition, the higher liver weight (Figure 1d) and body weight (Figure 1e) of the *db*/*db* mice were also consistent with the characteristics of NAFLD. Furthermore, *db*/*db* mice automatically suffered hyperglycemia, as shown by the significantly higher blood glucose than in the *bks* control group. Notably, when the blood glucose of mice exceeded the maximum of the glucometer (33.3 mmol/L), it was recorded as 33.3 mmol/L (Figure 1g). In addition, since hepatic ALT activity is approximately 3000 times higher than that of serum ALT activity [18], we mined data from our proteomic data. As shown in Table S2, the hepatic AST (*Got1*) and ALT (*Gpt2*) or ALP (*Alpl*) levels were significantly higher in *db*/*db* mice than in *bks* mice, by 96.7%, 41.7%, and 48.5%, respectively. Thus, liver damage occurred in the *db*/*db* mice group compared with the *bks* mice. Similarly, Liu et al. [19] have also found hepatic ALT and AST levels were slightly higher in the *db*/*db* mice compared with *bks* mice by approximately 50% and 20%, respectively. Taken together, these data show that *db*/*db* mice were constructed as genetic NAFLD mice models.

### 2.2. Validations of Data Filtering and Quality Control in the Serum Samples

To obtain high-quality analysis results, further data filtering is warranted for the procedure of database search analysis. The accuracy FDR of the spectrogram, peptide, and protein identification was set at 1%, and the identification protein must contain at least one unique peptide. Figure S2a shows the total number of identified peptides and proteins after data filtering of the serum samples. Specifically, the number of total spectra is 18,452, among which 17,251 spectra matched the theoretical secondary spectrum. Then, 4319 peptides were identified and 4171 unique peptides were analyzed from the matched peptides. Finally, 833 peptides were identified and 744 proteins were quantified by specific peptides. After the mass spectrometry data were searched, a series of quality controls were needed to meet the criteria. As presented in Figure S2b–f, the verifications of quality control include protein coverage distribution, peptide length distribution, tolerance distribution of parent ion mass, peptide number distribution, and protein molecular weight distribution. Specifically, in the shotgun (also called bottom-up) strategy, the mass spectrometry scans the peptides with higher abundance first. Figure S2b shows that the coverage of most proteins is below 30%. Therefore, there is a positive correlation between protein coverage and abundance in the serum sample. In addition, as for the peptide length distribution presented in Figure S2c, most of the peptides are distributed in 7–20 amino acids, which conforms to the general rule based on enzymatic hydrolysis and the high-energy collisional dissociation (HCD) fragmentation mode. Among them, peptides with fewer than seven amino acids could not generate effective sequence identification due to too few fragment ions. Peptides with more than 20 amino acids are not suitable for fragmentation by HCD due to their high mass and charge number. Thus, the distribution of peptide length identified by mass spectrometry met the requirements of quality control. Additionally, Figure S2d shows that the first-order mass error of most spectrograms is less than 10 ppm, which conforms to the characteristics of high-precision mass spectrometry and this result verified that the mass precision of the mass spectrometer is normal. On the other hand, Figure S2e indicates that most proteins correspond to two or more peptides. During quantification, a protein corresponding to multiple specific peptides (or corresponding to multiple spectrograms) is beneficial to increase the accuracy and credibility of quantitative results. Figure S2f also shows that the molecular weights of the identified proteins are evenly distributed at different stages. Overall, these data verify that the results of the filtering data in the serum samples meet the criteria of quality control.

### 2.3. Validations of Data Filtering and Quality Control in the Liver Samples

Similarly, the validations of data filtering and quality control were also completed in the liver samples. As shown in Figure S3a, the number of total spectra is 346,416, among which 89,023 spectra matched the theoretical secondary spectrum. Next, 43,912 peptides were identified, and 42,070 unique peptides were analyzed from the matched peptides. Finally, 5830 peptides were identified and 5809 proteins were quantified by specific peptides. As for the data quality control of the spectra in the liver samples shown in Figure S3b–f, similar to the results of the serum samples, they also conform to the criteria of protein coverage distribution, peptide length distribution, tolerance distribution of parent ion mass, peptide number distribution, and protein molecular weight distribution. Taken together, the results of the filtering data presented high quality in the liver samples.

### 2.4. Biological Repeatability of the Proteome

**Figure 2:**
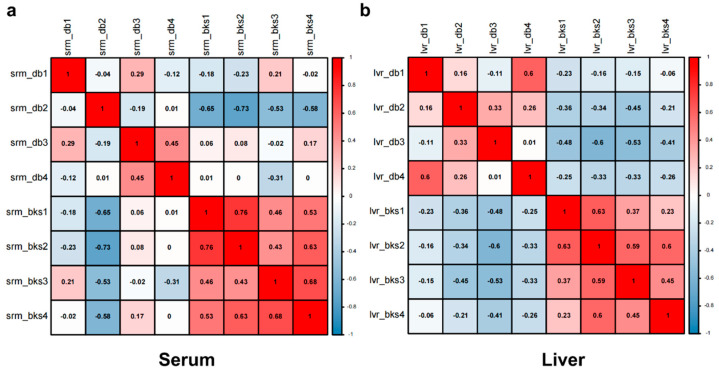
Sample repeatability analysis of quantitative serum total proteins for *bks* and *db*/*db* mice. Pearson’s correlation coefficients of the serum (**a**) and liver (**b**) represented the four repeats of *bks* mice distinguishing the repeats from *db*/*db* mice.

For biological duplicates, it is necessary to test whether the quantitative results of the biological duplicates are statistically consistent. Here, we used Pearson’s correlation coefficient as the statistical analysis method to evaluate the protein quantitative repeatability. The result is shown as a heat map drawn by calculating Pearson’s correlation coefficients between all samples. This coefficient is a value that measures the degree of linear correlation between two sets of data. Therefore, as indicated in Figure 2a,b, the two sets of Pearson’s correlation coefficients of both the serum and liver samples are closer to 0, which indicates that there is no correlation between each sample in the serum and the liver. Overall, these results reveal that both serum and liver total protein samples represent good quantitative reproducibility between their two groups.

### 2.5. Identification and Bioinformatic Analysis of Serum DEPs of db/db Mice

**Figure 3:**
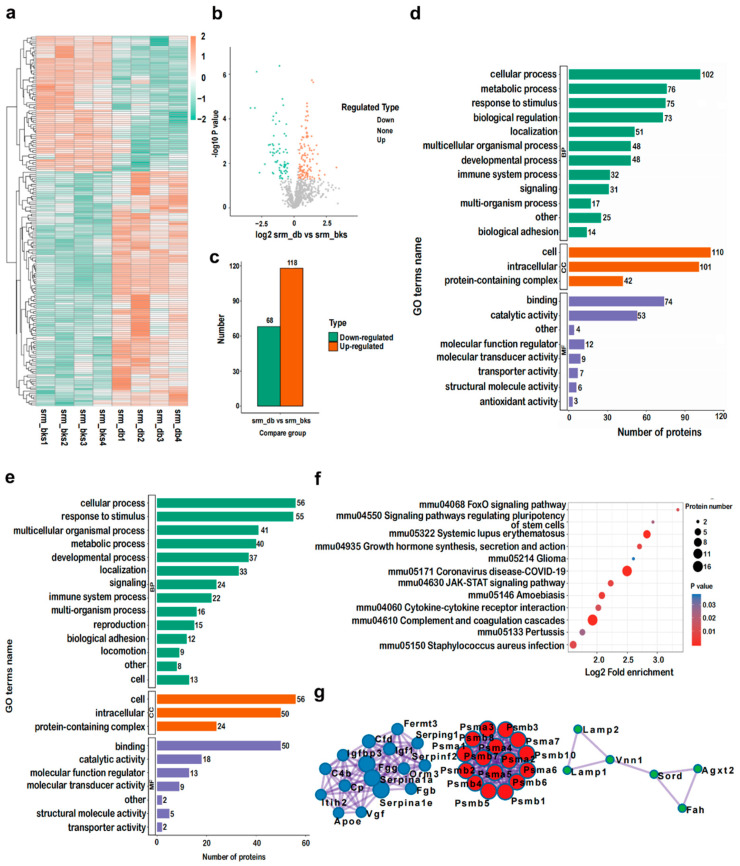
Identification and bioinformatics analysis of serum DEPs in *db*/*db* mice compared with the *bks* mice. (**a**) Heatmap, (**b**) volcano, and (**c**) histogram showing the distribution of serum DEPs in *db*/*db* mice compared with *bks* mice. GO (BP) analysis of (**d**) upregulated proteins and (**e**) downregulated serum proteins in *db*/*db* mice compared with *bks* mice. (**f**) KEGG pathway analysis of downregulated DEPs in the mouse serum. (**g**) PPI network of the optimized upregulated and downregulated DEPs in the mouse serum.

A total of 744 proteins were quantified from 833 identified proteins (Figure 3a). Finally, 186 DEPs, including 118 upregulated DEPs and 68 downregulated DEPs (quantified in all examined samples; ratio ≥ 1.3 or ratio ≤ 0.7, respectively, *p* < 0.05), were filtered for subsequent analysis (Figure 3b,c). Among both the upregulated and downregulated DEPs, GO biological process (BP) analysis indicated that these DEPs primarily engaged in the cellular process, metabolic process, response to stimulus, and biological regulation. GO cellular component (CC) analysis implied that these DEPs originated from the cell, intracellularly and protein-containing complexes. GO molecular function (MF) analysis revealed that these DEPs engaged in binding, catalytic activity, molecular function regulation, and molecular transducer activity, as well as antioxidant activity (Figure 3d,e). KEGG pathway analysis indicated that the downregulated DEPs in the serum of NAFLD models mainly enriched in the complement and coagulation cascades, coronavirus disease COVID-19, systemic lupus erythematosus, Staphylococcus aureus infection, amoebiasis, JAK-STAT signaling pathway, and FoxO signaling pathway.

As described in Figure 3g and Table 1, the protein-protein interaction (PPI) network revealed nine clusters including proteasomes that could classify the densely interconnected DEPs. These clusters were listed as follows: the regulation of insulin-like growth factor (IGF), transport and uptake by insulin-like growth factor binding proteins (IGFBPs), neutrophil degranulation, monocarboxylic acid metabolic process, small molecule catabolic process, plasma lipoprotein particle remodeling; protein-lipid complex remodeling; protein-containing complex remodeling. Moreover, the core proteins shown in Figure 3g are listed as follows: fibrinogen gamma chain (FGG), fibrinogen beta chain (FGB), insulin-like growth factor I (IGF1), proteasome subunit alpha type-2 (PSMA2), proteasome subunit beta type-7 (PSMB7), proteasome subunit beta type-8 (PSMB8), proteasome subunit beta type-6 (PSMB6), proteasome subunit beta type-2 (PSMB2), proteasome subunit alpha type-4 (PSMA4), proteasome subunit beta type-1 (PSMB1), apolipoprotein E (APOE), proteasome subunit beta type-10 (PSMB10), proteasome subunit alpha type-5 (PSMA5), proteasome subunit beta type-5 (PSMB5), proteasome subunit alpha type-6 (PSMA6), proteasome subunit alpha type-7 (PSMA7), alpha-1-antitrypsin 1–1 (SERPINA1A), proteasome subunit alpha type-1 (PSMA1), proteasome subunit beta type-4 (PSMB4), alpha-1-antitrypsin 1–5 (SERPINA1E), proteasome subunit beta type-3 (PSMB3), proteasome subunit alpha type-3 (PSMA3), complement C4-B (C4B), fermitin family homolog 3 (FERMT3), serine (or cysteine) peptidase inhibitor, clade G, member 1 (SERPING1), alpha-1-acid glycoprotein 3 (ORM3), complement factor D (CFD), inter-alpha-trypsin inhibitor heavy chain H2 (ITIH2), integrin beta-2 (ITGB2), and insulin-like growth factor-binding protein 3 (IGFBP3).

### 2.6. PRM Validations of Serum DEPs

It has been reported that PRM verification is more authentic in reflecting the quantification of the DEPs than that of Western blotting or immunofluorescence [13]. To verify the DEPs generated from the TMT-labeling proteomic analysis in the serum samples, a series of upregulated and downregulated DEPs were selected to conduct further PRM validations, and the results are shown in Table 2. These results showed that the PRM fold changes of these selected DEPs were consistent with the global proteomic fold changes, which validated the reliability and accuracy of the TMT-labeling proteomic analysis in the serum samples.

### 2.7. Identification and Bioinformatic Analysis of Hepatic DEPs in db/db Mice

**Figure 4:**
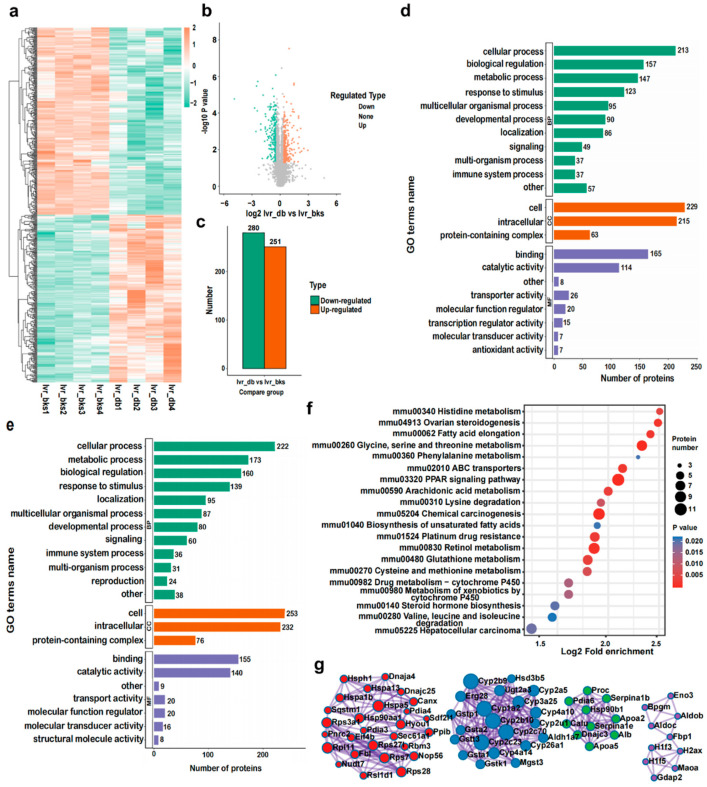
Identification and bioinformatics analysis of hepatic DEPs in *db*/*db* mice compared with *bks* mice. (**a**) Heatmap, (**b**) volcano, and (**c**) histogram depicting the distribution of the hepatic DEPs in *db*/*db* mice compared with *bks* mice. GO (BP) analysis of (**d**) the upregulated proteins and (**e**) downregulated hepatic proteins in *db*/*db* mice compared with *bks* mice. (**f**) KEGG pathway analysis of upregulated DEPs in the mouse liver. (**g**) PPI network of the optimized upregulated and downregulated DEPs in the mouse liver.

A total of 5609 proteins were quantified from 5830 identified proteins in the liver tissues. Among these proteins, 280 downregulated and 251 upregulated DEPs (quantified in all examined samples; ratios ≥ 1.3 or ratios ≤ 0.7, respectively, *p* < 0.05) were selected for further analysis (Figure 4a–c). GO (BP) analysis indicated that the upregulated DEPs (Figure 4d) and downregulated DEPs (Figure 4e) were involved in processes including cellular, biological regulation, metabolic, response to stimulus, multicellular organismal, and localization, signaling, multi-organism process, and immune system process. Additionally, the GO (CC) analysis showed that the DEPs existed principally in cells and intracellular cells or protein-containing complexes. GO (MF) analysis indicated that the DEPs engaged primarily in binding, catalytic activity, transporter activity, molecular function regulator, transcription regulatoractivity, molecular transducer activity, and antioxidant activity. In addition, KEGG pathway analysis revealed that the upregulated DEPs mostly participated in the PPAR signaling pathway, chemical carcinogenesis, retinol metabolism, glycine, serine, and threonine metabolism, fatty acid elongation, biosynthesis of unsaturated fatty acids, glutathione metabolism, steroid hormone biosynthesis, hepatocellular carcinoma, arachidonic acid metabolism, and drug metabolism-cytochrome P450 (Figure 4f). Figure 4g and Table 3 indicate the core DEPs were identified in 14 clusters. The clusters include protein folding, ER-localized multiprotein complex, protein processing in the endoplasmic reticulum, chemical carcinogenesis, retinol metabolism, post-translational protein phosphorylation, and the regulation of insulin-like growth factor (IGF) transport as well as the uptake by insulin-like growth factor binding proteins (IGFBPs), plasma lipoprotein remodeling, glycolysis/gluconeogenesis, neutrophil degranulation, and the synthesis and secretion as well as the deacylation of ghrelin, fatty acid metabolism, glycosylation, steroid hormone biosynthesis, androgen metabolic process, endocytosis, plus the terminal pathway of complement. Moreover, these interconnected DEPs are listed as follows: protein disulfide-isomerase A6 (PDIA6), DnaJ homolog subfamily C member 3 (DNAJC3), heat shock protein 5 (HSPA5), heat shock protein 90, beta (Grp94), member 1 (HSP90B1), cytochrome P450 1A2 (CYP1A2), albumin (ALB), UDP-glucuronosyltransferase 2A3 (UGT2A3), cytochrome P450 2B10 (CYP2B10), cytochrome P450 3A25 (CYP3A25), protein transport protein Sec61 subunit alpha isoform 1 (SEC61A1), cytochrome P450 2B9 (CYP2B9), hypoxia upregulated protein 1 (HYOU1), mesencephalic astrocyte-derived neurotrophic factor (MANF), calreticulin (CALR), calnexin (CANX), cytochrome P450, family 2, subfamily c, polypeptide 23 (CYP2C23), NADPH-dependent 3-keto-steroid reductase Hsd3b5 (HSD3B5), heat shock protein HSP 90-alpha (HSP90AA1), cytochrome P450 2C70 (CYP2C70), aldehyde dehydrogenase family 1, subfamily A7 (ALDH1A7), cytochrome P450 family 51 subfamily A member 1 (CYP51A1), farnesyl diphosphate farnesyl transferase 1 (FDFT1), protein disulfide isomerase associated 4 (PDIA4), 7-dehydrocholesterol reductase (DHCR7), caspase-3 (CASP3,) methylsterol monooxygenase 1 (MSMO1), epidermal growth factor receptor (EGFR), 3-hydroxy-3-methylglutaryl-Coenzyme A synthase 1 (HMGCS1), apolipoprotein A-V (APOA5), DnaJ homolog subfamily B member 11 (DNAJB11), translocon-associated protein subunit gamma (SSR3), farnesyl pyrophosphate synthase (FDPS), cytochrome P450 family 17 subfamily A member 1 (CYP17A1), cytochrome P450 2A5 (CYP2A5), 3-hydroxy-3-methylglutaryl-coenzyme A reductase (HMGCR), apolipoprotein A-II (APOA2), cytochrome P450 4A10 (CYP4A10), NAD(P) dependent steroid dehydrogenase-like (NSDHL), heat shock 70 kDa protein 1B (HSPA1B), mevalonate (diphospho) decarboxylase (MVD), glutathione S-transferase kappa 1 (GSTK1), minor histocompatibility antigen H13 (HM13), mevalonate kinase (MVK), cytochrome P450 4A14 (CYP4A14), cytochrome P450 2D9 (CYP2D9), 60S ribosomal protein L11 (RPL11), glutathione S-transferase theta-3 (GSTT3), 40S ribosomal protein S3a (RPS3A), alpha-1-antitrypsin 1-2 (SERPINA1B), cytochrome P450 26A1 (CYP26A1), alpha-1-antitrypsin 1–5 (SERPINA1E), 40S ribosomal protein S7 (RPS7), alpha-1-acid glycoprotein 2 (ORM2), isopentenyl-diphosphate delta-isomerase 1 (IDI1), peptidyl-prolyl cis-trans isomerase B (PPIB), vitamin K-dependent protein C (PROC), 40S ribosomal protein S28 (RPS28), glutathione S-transferase A2 (GSTA2), glutathione S-transferase A1 (GSTA1), protein disulfide-isomerase A3 (PDIA3), signal sequence receptor, delta (SSR4), 17-beta-hydroxysteroid dehydrogenase type 6 (HSD17B6), thioredoxin domain-containing protein 5 (TXNDC5), signal peptidase complex catalytic subunit SEC11C (SEC11C), cytochrome P450 2U1 (CYP2U1), glutathione S-transferase P 1 (GSTP1), and calumenin (CALU) (Figure 4g).

### 2.8. PRM Validations of Hepatic DEPs

Likewise, to verify the DEPs generated from the TMT-labeling proteomic analysis in the liver samples, we arbitrarily selected some upregulated and downregulated DEPs for further PRM validations. The results show that the PRM fold changes of these selected DEPs were completely concordant with the global proteomic fold changes (Table 4), which proved the reliability and accuracy of the TMT-labeling proteomic analysis in the liver samples.

### 2.9. The Confirmation of Common DEPs in the Serum and Liver of db/db Mice

**Figure 5:**
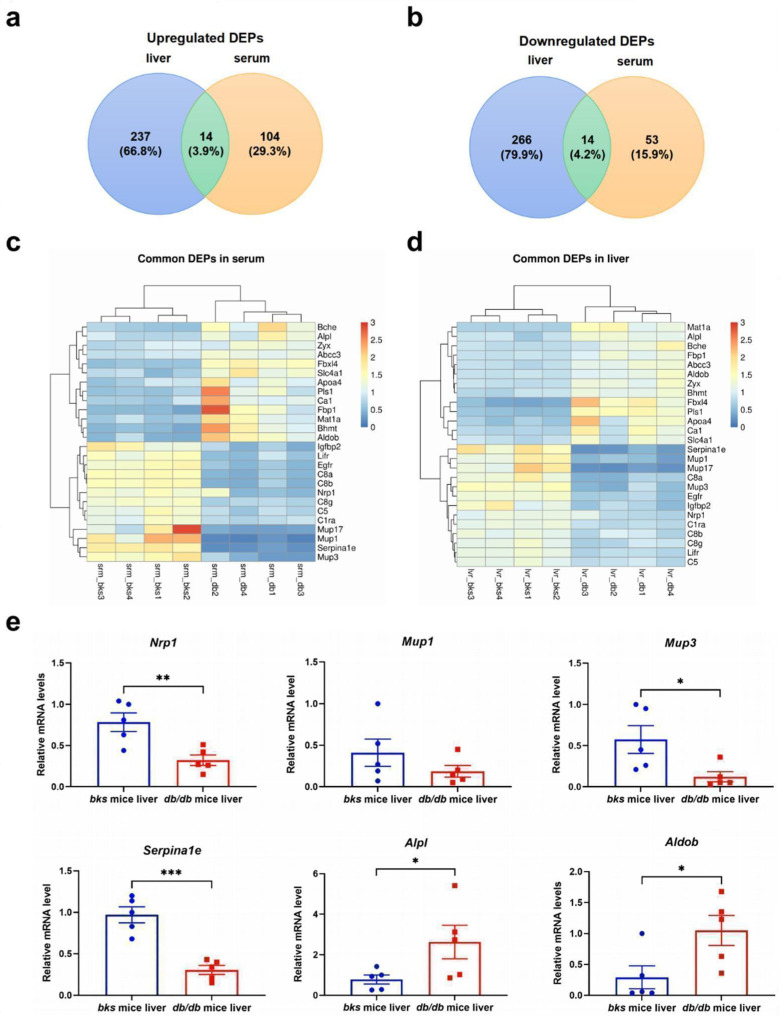
The confirmation of common DEPs in the sera and livers of *db*/*db* mice. (**a**) Commonly up-regulated DEPs in the sera and livers of *db*/*db* mice and *bks* mice (*n* = 5). (**b**) Commonly downregulated DEPs in the sera and livers of *db*/*db* mice and *bks* mice (*n* = 5). (**c**) Heatmap of the commonly upregulated DEPs. (**d**) Heatmap of the commonly downregulated DEPs. (**e**) The validations of some of the common DEPs. Red dots represent *bks* mice and blue squares represent *db/db* mice. * *p* < 0.05; ** *p* < 0.01; *** *p* < 0.001.

Because proteins secrete from the liver and are released into the blood they may accurately represent the status of liver injury or steatosis when detected in the liver and serum [9], the validation of commonly expressed proteins in the liver and serum benefits the discovery of NAFLD biomarkers. Among the DEPs in the serum and liver samples of *db*/*db* mice and the control group, except for 2 uncharacterized proteins, we found 13 commonly upregulated DEPs and 13 downregulated DEPs (Figure 5a,b). Figure 5c,d show the heatmap of common upregulated DEPs in the serum and the liver, the proteins are listed as follows: methionine adenosyltransferase 1A (MAT1A), alkaline phosphatase (ALPL), butyrylcholinesterase (BCHE), fructose-1,6-bisphosphatase 1 (FBP1), canalicular multispecific organic anion transporter 2 (ABCC3), fructose-bisphosphate aldolase B (ALDOB), zyxin (ZYX), betaine (BHMT), F-box/LRR-repeat protein 4 (FBXL4), plastin-1 (PLS1), apolipoprotein A-IV (APOA4), carbonic anhydrase 1 (CA1), and solute carrier family 4 (anion exchanger), member 1 (SLC4A1). Additionally, the commonly downregulated DEPs are listed as follows: alpha-1-antitrypsin 1–5 (SERPINA1E), major urinary protein 1 (MUP1), major urinary protein 17 (MUP17), major urinary protein 3 (MUP3), epidermal growth factor receptor (EGFR), insulin-like growth factor-binding protein 2 (IGFBP2), neuropilin-1 (NRP1), leukemia inhibitory factor receptor (LIFR), complement C1r-A (C1RA), complement C5 (C5), complement component C8 alpha chain (C8A), complement component C8 beta chain (C8B), and complement component C8 gamma chain (C8G). Moreover, as core commonly DEPs in the liver and serum, the downregulated DEPs, such as NRP1, MUP3, SERPINA1E, and IGFBP2, and upregulated DEPs, such as ALPL and ALDOB were verified at the mRNA level in the liver samples by qPCR. The results show that the mRNA levels of *Nrp1*, *Mup3*, and *Serpina1e* were significantly downregulated whereas *Alpl* and *Aldob* were significantly upregulated in the *db*/*db* mice compared with the *bks* mice group (Figure 5e). To summarize, these results suggest that the trend of mRNA expression was consistent with that of proteomic analysis among the commonly regulated DEPs.

## 3. Discussion

Linked to obesity, type 2 diabetes, and other metabolic dysregulation, the continuously increased incidence of NAFLD has become a worldwide public health problem [20]. Sustainable efforts should discover candidate biomarkers for NAFLD diagnosis and prognosis. Recently, TMT-labeled proteomic analysis has been considered a valuable tool for the diagnosis and prognosis of diseases. As a classic genetically obese mice model, *db*/*db* mice presented obesity and NAFLD, making the *db*/*db* mouse the optimal animal model for NAFLD. Of note, instead of the costly and not widely acceptable liver biopsy, there is an urgent need to discover non-invasive serum biomarkers for NAFLD and the development of its metabolic complications. Herein, we performed two sets of TMT-labeled proteomic analyses based on the sera and livers of *db*/*db* mice. Although we have identified plenty of candidate biomarkers in the form of DEPs, there were only 26 DEPs expressed in both the serum and liver. The protein levels and mRNA levels of five DEPs showed the same trend, on the one hand, the downregulated DEPs were neuropilin-1 (NRP1), major urinary protein 3 (MUP3), and alpha-1-antitrypsin 1-5 (SERPINA1E), and on the other hand, the upregulated DEPs were alkaline phosphatase (ALPL) and fructose-bisphosphate aldolase B (ALDOB).

As a transmembrane glycoprotein, NRP1 exists in non-parenchymal liver cells such as hepatic stellate cells (HSCs) and liver sinusoidal endothelial cells (LSECs). NRP1 is related to axonal activation, angiogenesis, and the increased level of NRP1 in the hepatocyte is related to hepatocellular carcinoma. Furthermore, NRP1 plays an essential role in HSC activation in the liver. Specifically, NRP1 is a co-receptor of platelet-derived growth factor (PDGF) and transforming growth factor-β (TGF-β), and in vitro studies have shown that HSC activation upregulates *Nrp1* mRNA levels. In contrast, NRP1*1* upregulated the smad2/3 signaling pathway to activate fibroblast cells and promote liver fibrosis [21]. Moreover, dependent inhibition of NRP1 targeted HSCs and ameliorated alcohol-induced steatohepatitis by decreasing hepatic lipid droplets as well as inflammation through regulation of the IGFBP3 and SERPINA1A12 signaling pathways [22]. Since HSC activation is the central role of liver fibrosis, NRP1 is a potential therapeutic target for rescuing liver fibrosis [23]. In our study, however, NRP1 mRNA and protein levels were downregulated in the livers of *db*/*db* mice, which was beyond our anticipation. According to animal research based on *db*/*db* mice, we found that advanced glycation ending products (AGEs) reduced NRP1 levels in the kidney. Thus, we reasonably hypothesized that the inhibition of NRP1 in the *db*/*db* mice might be due to exposure to accumulated AGEs in the liver [24,25,26,27].

Major urine proteins (MUPS) belong to the lipocalin superfamily produced in the liver and excreted into the urine [28]. There is only one kind of MUP in humans [29], whereas in mice, there are 21 genes and 21 pseudogenes for MUPs, and they play crucial roles as pheromones in male mice to attract females and scent-mark [30,31,32]. MUP1 and MUP2 have been previously regarded as the regulators of glucose and lipid metabolism [33,34,35,36], whereas in our study, as a newly identified downregulated protein in both the *db/db* mouse liver and serum, the role of MUP3 in NAFLD is unclear. It has been reported that MUPs are associated with circadian rhythm, and we therefore speculate MUP3 might have the same functionality in the mice [37]. However, to have a better understanding of MUPs’ functions, further studies might use an all*-Mups*-gene-knockout mouse model based on the CRISPR-cas9 technique [38].

Anti-protease alpha 1-antitrypsin is primarily expressed in hepatocytes and secreted into the bloodstream to protect the lung from proteolytic degradation with neutrophil elastases [39,40]. In humans, there is only one type of alpha 1-antitrypsin encoded by *SEPRPINA1*, whereas there are five types of alpha 1-antitrypsin in mice with a C57BL/6J background, among which alpha 1-antitrypsin 5 is encoded by the *Serpina1e* gene [41]. Here, we identified that the *Serpina1e* gene was downregulated in the *db*/*db* mouse liver, and α1-antitrypsin 5 decreased in the *db*/*db* mouse liver and serum. In addition, a previous study has reported that alpha 1-antitrypsin 5 could be downregulated in high-fat-diet-induced NAFLD mice sera while preventive exercise could restore the serum level of alpha 1-antitrypsin 5 [42]. Furthermore, the supplementation of human alpha 1-antitrypsin in mice fed alcohol could ameliorate the accumulation of intrahepatic lipid droplets and body weight [43]. Hence, alpha 1-antitrypsin might be a biomarker and therapeutic target for NAFLD.

As for the upregulated DEPs, alkaline phosphatase (ALPL), also named ALP, together with alanine aminotransferase (AST), aspartate aminotransferase (ALT), bilirubin, and albumin is already known as a biochemical indicator for liver function tests. If the serum level of ALP exceeds the normal range of 30–120 IU, it means that liver function is impaired [44]. Apart from the aforementioned information, ALDOB is enriched in the liver and kidney, as well as the small intestine [45]. ALDOB is a glycolytic enzyme that regulates fructose catabolism, playing essential roles in gluconeogenesis and lipogenesis [9]. Nesteruk et al. [46] also discovered that the hepatic and serum ALDOB level was upregulated among the high-fat-induced NAFLD mice compared with regular-diet-fed mice. Moreover, Niu et al. [9] found that plasma ALDOB protein levels were elevated in both NAFLD patients and high-fat-diet-induced NAFLD mice, and this trend conformed to that of our results. They speculated that the increase of ALDOB results from leakage because of the excessive fat accumulation in the hepatocyte. Mutations of the human *Aldob* gene could cause defective fructose metabolism and this disease is called hereditary fructose intolerance (HFI), patients with HFI could rapidly develop NAFLD and fibrosis with very low levels of fructose, and are prone to suffer from liver and kidney dysfunctions, especially among infants [47,48,49]. To mimic human HFI phenotypes, a global knockout of the *Aldob* gene in mice exposed to fructose rapidly developed into hepatic steatosis and inflammation, whereas these conditions could be rescued with pharmacological inhibition of ketohexokinase (KHK), an enzyme involved in the pathway of fructose metabolism [50,51].

Apart from the aforementioned identified DEPs by qPCR, we also compared the variations of the rest of the common upregulated and downregulated DEPs in human serum originating from a series of NAFLD cohorts reported in the literature. The information on the upregulated DEPs was as follows. Serum methionine adenosyltransferase 1A (MAT1A) levels and hepatic *Mat1a* gene expressions were downregulated in NAFLD patients compared with healthy control [52,53,54]. Serum butyrylcholinesterase (BCHE) activity was significantly increased in NAFLD patients compared with controls [55]. ATP-binding cassette subfamily C member 3 (ABCC3) protein level was upregulated in the liver samples of NASH but not NAFL patients compared with healthy controls [56]. Betaine (BHMT) levels in the blood were inversely associated with the severity of NAFLD in humans [57], and many studies suggest a preventive role of betaine in NAFLD [58,59]. Zyxin (ZYX) is a focal-adhesion-associated phosphoprotein involved in cell motility, cell migration, and infiltration by acting on the actin cytoskeleton [60]. Zyxin promotes cell dissemination as part of the integrin signaling pathway [61]. Yet to our knowledge, there was no investigation into the relationship between zyxin and NAFLD. F-Box and leucine reach protein 4 (FBXL4) are mitochondria-related genes that exert effects on mitochondrial DNA stabilization and bioenergetics, but to date, to our knowledge, there is no study comparing the serum FBXL4 levels among NAFLD patients and healthy people [62]. Plastin1 (PLS1), together with PLS2 and PLS3, are actin-bundling proteins. PLS1 plays a critical role in influencing cell functions such as cytoskeleton maintenance and cell-cell adhesion, as well as cell migration [63]. Zhang et al. [64] have reported that PLS1 protein levels were elevated in colorectal cancer. However, there was no study identifying the serum level of PLS1 in NAFLD patients. Apolipoprotein A-IV (APOA4) is a lipid-binding protein that engages in lipid regulation and glucose metabolism [65]. Several NAFL patient cohorts have revealed the upregulation of APOA4 expression in the steatotic liver [66] during the early stages of liver fibrosis [67] and elevated plasma levels of APOA4 among [9] NAFL patients. Carbonic anhydrase 1 (CA1) [68] is a member of the carbonic anhydrase family that reversibly catalyze hydrated CO_2_ into HCO_3_^−^, which then directly binds to carbo calcium ions to form calcium carbonate [69]. Yuan et al. found that CA1 was overexpressed in the calcified human and mouse aortic stenosis tissues and that inhibiting CA1 expression could be a potential therapeutic target for aortic stenosis [70]. Moreover, carbonic anhydrase is also involved in biosynthetic processes like lipogenesis, ureagenesis, and gluconeogenesis [68], but its role in NAFLD is unknown. Solute carrier family 4 (anion exchanger), member 1 (SLC4A1) is a component of the erythrocyte ghost membrane that plays an important part in mediating Cl^−^/HCO_3_^−^ exchange in the blood [71]. SLC4A1 is distributed mainly in erythrocytes, intercalated cells of the renal collecting duct, heart, and colon, and has an association with a series of diseases such as hemolytic anemia and distal renal tubular acidosis [72]. Furthermore, SLC4A1 was also relevant to lipid peroxidation and the reduction of the GSH/GSSG ratio in diabetes mellitus [73]. However, to our knowledge, so far its role in NAFLD is unclarified.

As for the common downregulated DEPs in the liver and serum of our mouse model, the information on relevant human serum levels are as follows. Epidermal growth factor receptors (EGFRs) play a key role in hepatocyte proliferation, liver regeneration, and hepatocellular carcinoma. EGFR inhibition attenuated steatosis by regulating key transcription factors regulating fatty acid synthesis and lipolysis in NAFLD mouse models [74]. Hortet et al. [75] implied that there was a significant inverse correlation between hepatic EGFR expression and hepatic steatosis levels in liver biopsies from obese patients with varying degrees of steatosis. However, Giraudi et al. [76] revealed that plasma EGFR levels showed no significant reduction after weight-loss surgery compared with that pre-surgery, although clinical biochemical parameters (BMI, HbA1c, and HOMA-IR) returned to the normal range. Leukemia inhibitory factor receptor (LIFR or LIFRβ) is the receptor for leukemia inhibitory factor (LIF), a family member that belongs to the interleukin (IL)-6 cytokine [77,78]. Yuan et al. [79] found that serum LIF levels in NAFLD patients were higher than that of non-NAFLD subjects. Moreover, they found that LIF attenuated liver steatosis via binding to LIFR and activating the STAT3 pathway, which provided a rationale for LIF–LIFR to be a potential therapeutic target for NAFLD treatment. Insulin-like growth factor-binding protein 2 (IGFBP2) is one of six proteins that bind to insulin-like growth factor (IGF) and exert influence on regulating glucose and lipid metabolism [80]. Stanley et al. [81] discovered a negative association between the hepatic IGFBP2 mRNA levels and the grades of liver steatosis in NAFLD patients. In addition, Fahlbusch et al. [82] and Yang et al. [83] reported that circulating IGFBP2 levels were lower among obese NAFLD patients compared with that of healthy controls whereas weight loss restored the plasma IGFBP2 level accompanied by a downregulation of fatty liver contents [82]. A recent study has also indicated that hepatic IGFBP2 mRNA levels were lower in NASH patients compared with healthy subjects [80]. These results suggest IGFBP2 is a potential novel non-invasive biomarker for NAFLD. Complement C8, a secreted protein, is a crucial component of the membrane attack comprised of *C8a*, *C8b*, and *C8g*. Hou et al. [84] discovered that NASH patients have lower serum *C8g* levels than healthy controls. As for serum complement C5, Hu et al. [85] revealed that serum C5 levels were associated with NAFLD and Hillebrandt et al. [86] found that C5 is a causal effector of liver fibrosis. Complement C1R is a subcomponent of the serine proteinase C1 that plays a prominent role in the classical pathway of the complement system [87]. Nevertheless, to our knowledge, there was no direct evidence of the serum C1R level in NAFLD patients.

PRM validation revealed a series of proteins of interest such as stromal cell-derived factor (SDF2L1), 17-beta-hydroxysteroid dehydrogenase type 2 (HSD17B2), and 17-beta-hydroxysteroid dehydrogenase type 6 (HSD17B6). In humans, *Sdf2l1* and *Sdf* are paralogous genes of the O-mannosyltransferase family whereas mouse SDF2L1 and SDF protein sequences share 78% similarity and 68% identity [88]. *Sdf2l1* and *Sdf* are widely expressed in the liver and kidney [89]. Both human and mouse *Sdf2l1* are located in the endoplasmic reticulum (ER) in the form of components of a multiprotein complex including BiP/GRP78 (binding immunoglobulin protein or glucose-related protein 78) and GRP94 (glucose-related protein 94) [90]. Additionally, *Sdf2l1* participates in the process of protein transportation across the ER and protein folding, and also interacts with antimicrobial peptides and thus plays an essential role in innate immunity [91,92]. Chronic ER stress has a close association with diabetes [93] and NAFLD [94]. Schott et al. [95] implied that the SDF2L1 protein level was upregulated under ER stress whereas silencing SDF2L1 exacerbated ER stress and the unfolded protein response [88]. Thus, the SDF2L1 protein plays a critical role in ER stress. Additionally, in our proteomic analysis and PRM validations, the *Sdf2l1* protein level was unexpectedly downregulated among *db*/*db* mice compared with *bks* mice. This phenomenon is similar to the research of Sasako et al. who found that *Sdf2l1* gene and protein levels were downregulated, accompanied by the suppression of other ER stress inducers such as XBP1, either in fasting or refeeding conditions, which was presumably due to insufficient activation of ER stress for further triggering of excessive ER stress [94]. Moreover, Sasako et al. [94] also found that *Sdf2l1* interacts with ER-associated degradation-related protein, transmembrane emp24-like trafficking protein 10 (TMED10), and suppression of *Sdf2l1* in the liver exacerbated insulin resistance and hepatic steatosis. The restoration of *Sdf2l1* reversed these aforementioned effects. In addition, among patients with diabetes, insufficient induction of *Sdf2l1* has a positive correlation with the progression of insulin resistance and steatohepatitis. Therefore, *Sdf2l1* could become a potential therapeutic target and sensitive biomarker for diabetes and NAFLD.

Unlike HSD3B5, a protein only expressed in mice, both HSD17B2 and HSD17B6 are expressed in humans and mice. Specifically, HSD17B2 is expressed in a wide variety of tissues, such as the breast, uterus, prostate, placenta, liver, and kidney, and can catalyze enzymatic reactions of both C18- and C19-substrates [96]. On the other hand, HSD17B6 is predominantly distributed in the liver, lung, and prostate, and can convert 3α-androstanediol to dihydrotestosterone (DHT), the most potent form of androgen [97,98]. Chan et al. demonstrated that dysregulation of DHT could affect the progression of prostate cancer and breast cancer [99]. Our proteomic analysis and PRM validation confirmed that HSD17B2 and HSD17B6 were both significantly downregulated in *db*/*db* mice compared with *bks* mice, which could provide insights into the association between HSD17B2 or HSD17B6 and NAFLD and diabetes based on steroid metabolism. Of note, to get a better understanding of how the major upregulated and downregulated DEPs connect to NAFLD, we summarized a graphical pathway in Figure S4.

Although we have differentiated ample potential hepatic and serum total proteins of the *db*/*db* mouse from healthy controls, there is still a long way to go to translate into clinical evidence for validating effective and accurate non-invasive biomarkers for NAFLD patients. Therefore, it is urgent to perform serum or plasma proteomic analysis relying on extensive independent clinical NAFLD cohorts.

Study Limitations

Considering the homology of proteins between mice and humans, some DEPs that showed potential for becoming non-invasive biomarkers in *db*/*db* mice might not take effect in humans. For example, the *Serpina1e* and *Mup3* genes do not exist in humans. To avoid the divergence of homologs among mice and humans, further experiments should focus on comparing the DEPs in mice with that of NAFLD patient cohorts. Furthermore, to strengthen our findings for clinical purposes, patient cohorts related to obesity with NAFLD and diabetes will be developed in the future. Of note, in the current study, we used the total serum and liver proteins for proteomic analysis and the subsequent confirmation. However, to give precise gene or protein expression, isolated primary hepatocytes should be utilized in future experiments.

## 4. Methods

### 4.1. Animals

Six-week-old male *db*/*db* mice and age-matched male *bks* mice were purchased from GemPharmatech Co., Ltd. (Nanjing, China). The *db*/*db* mice and *bks* mice were fed a chow diet for 12 weeks and sacrificed to collect the liver and serum samples. The animal ethics committee of the University of Science and Technology of China (USTC) approved the animal protocols (USTCACUC212401038).

### 4.2. Sample Collections

After 12 weeks, the mice were anesthetized and sacrificed. PBS solution was perfused from the apex of the mice’s hearts. Blood was withdrawn retro-orbitally and placed for 1 h at room temperature and then the serum was extracted from the supernatant of blood centrifuged at 1000× *g* for 10 min at 4 °C. The liver samples were removed and transferred to liquid nitrogen following the sacrification of the mice. The left liver lobe and serum were transferred to a −80 °C refrigerator before use and the right liver lobe was fixed with 4% paraformaldehyde for 24 h and then sucrose solution at 4 °C followed by dehydration with 30% for 24 h. The remaining liver tissues were re-stored in liquid nitrogen.

### 4.3. Oil Red O Staining

The liver tissues were firstly dehydrated with 30% sucrose solution at 4 °C and subsequently embedded and sliced into 8 μm serial sections at −23 °C. After quickly soaking with 60% isopropanol, the areas were dyed with a 60% Oil Red O (ORO) dye mixture (Poly Scientific R&D Corp., Cleveland, NY, USA) for 1 min and washed with 60% isopropanol and subsequently with ddH_2_O. Afterward, the sections were sealed with gelatin glycerin. ORO staining images were observed under the microscope.

### 4.4. The Extraction and Digestion of Proteins

The liver and serum samples were differently processed. Briefly, the prepared lysis buffer was made of 8 m urea mixed with a 1% protease inhibitor cocktail (Sigma, St. Louis, MO, USA). The liver samples were completely ground into powder in liquid nitrogen and mixed with liquid, 4 volumes of lysis buffer, and the powder was sonicated on ice three times with a high-strength ultrasonic processor (Scientz, Ningbo, China). Then the supernatants were collected after removing the unresolved fragments by centrifugation at 12,000× *g* for 10 min at 4 °C. On the other hand, to remove the cell debris and collect the supernatant, the serum samples were centrifuged at 12,000× *g* for 10 min at 4 °C. The highly abundant proteins were removed using Seppro ^®^ MouseSpin Columns kit (Sigma) and the concentration of the protein was assessed using the BCA Protein Assay Kit (Sigma, St. Louis, MO, USA). Then the protein solution was diluted with 100 mM TEAB followed by reduction and alkylation (5 mM dithionite at 56 °C for 30 min and 11 mM iodoacetamide for 15 min in darkness at room temperature). The samples were then digested, firstly at a 1:50 trypsin to protein mass ratio overnight and secondly at a 1:100 trypsin to protein mass ratio at 37 °C for 4 h.

### 4.5. TMT Labeling, HPLC Separation, and LC-MS/MS Analysis

The peptide mixtures were firstly pooled, desalted by using Strata X C18 SPE columns (Phenomenex, Torrance, CA, USA), and then dried by vacuum centrifugation. After being reconstituted in 0.5 M TEAB, these peptide mixtures were processed with a TMT/iTRAQ Kit (Thermo Fisher Scientific, Shanghai, China). Specifically, after defrosting and dissolving in acetonitrile, the peptides were mixed with the labeled reagent and incubated for 2 h at room temperature, then the mixture was desalted and freeze-dried in a vacuum. For HPLC grading, the peptides were separated by high pH reversed-phase HPLC on an Agilent 300Extend C18 column. Briefly, the grading gradient of the peptides was 8–32% acetonitrile, pH 9.60 components were separated in 60 min. For the subsequent liquid chromatography-mass spectrometry analysis, the peptides were dissolved in liquid chromatography mobile phase A, separated by EASY nLC 1000 ULTRA high-performance liquid system and separated by an ultra-performance liquid phase system, and then injected into an NSI ion source for ionization as well as analysis based on Q Exactive Plus mass spectrometry.

### 4.6. Database Searches

The retrieval parameter of the secondary mass spectrometry (MS) data based on Maxquant (V1.6.15.0, Berlin, Germany) created by Max-Planck institute of biochemistry, computational systems biochemistry, which was set as follows. Firstly, a reverse database of Mus_musculus_10090_SP_20201214. Fasta (17,063 sequences) was applied to evaluate the false positive rate (FDR). In addition, to remove the effects of the contamination of proteins on the identified results, the contamination database was supplemented in this experiment. The digestion mode was set to Trypsin/P and the number of the missing tangent position was set to 2. Moreover, the minimum peptide length was set to 7 amino acid residues and the maximum modification of the peptide was set to 5. In addition, the mass error tolerance of the primary parent ions of the first and main search was set at 20 PPM and 4.5 PPM, respectively. Furthermore, the secondary fragment ions were set at 20 PPM. Importantly, for further data filtering, the accuracy of FDR of the spectrogram, peptide, and protein identification was set at 1%. The identification protein must contain at least one unique peptide.

### 4.7. Bioinformatics Analysis

Differentially expressed proteins (DEPs) between two groups were identified using a 1.3-fold change and a *p*-value of less than 0.05 as the thresholds based on the *t*-test. We performed multiple types of enrichment analysis such as gene ontology (GO) classification and the Kyoto encyclopedia of genes and genomes (KEGG) pathway for the annotation of DEPs. GO is an important tool for bioinformatics analysis that can describe the abundant properties and characteristics of genes and their products. GO annotations can be classified into three categories: biological process (BP), cellular component (CC), and molecular function (MF). KEGG makes a connection to known molecular interactions such as metabolic pathways, complexes, and biochemical reactions. Cluster analysis was utilized to detect the correlation among the DEPs based on the GO classification, KEGG pathway, and protein domain enrichment. Besides this, protein-protein interaction (PPI) analysis was performed through the STRING (V.11.0, Zurich, Switzerland) database created by the University of Zurich, and Cytoscape (V.3.8.1, Boston, MA, USA ) software created by Cytoscape consortium was adopted to present the network. In addition, we also employed ClusterViz (V. 1.0.3, Bochum, Germany) originating from Ruhr-Universität Bochum [100] embedded in Cytoscape to conduct clustering analysis based on the molecular complex detection (MCODE) algorithm [101], of which the criteria were set as degree cut-off as 2, node score cut-off as 0.2, max depth as 100 k-score as 2, and high confidence was set as no less than 0.7 [102]. Furthermore, primary component analysis (PCA), heatmap, bubble diagram, and Venn diagram were analyzed using the R package in version 3.5.2 [103,104].

### 4.8. Parallel Reaction Monitoring (PRM) Validation

Owing to the high-resolution and high-precision mass spectrometry (MS), PRM can selectively detect the target protein and targeted peptide (such as post-translational modification of the peptide), thus achieving the absolute quantification of the target protein/peptide. Here, we randomly selected 10 serum DEPs and 15 hepatic DEPs to further conduct PRM validation through PRM-MS analysis based on PTM BioLabs (Jingjie, China). The characteristic peptide of the target protein was identified and only the unique peptide sequence was selected for the subsequent PRM. Sixty micrograms of these peptides were prepared following the TMT analysis protocol. PRM was processed on an LC/MS-MS system and parameters were set as follows, isolation width: 0.7 *m*/*z*; maximum injection time: 100 ms; and collision energy: 30% [100,105]. Of note, the MS measurements were performed on the Q-Exactive HF MS for the liver samples and Serum Q-Exactive Plus for the serum samples, separately. The obtained PRM-MS raw data were treated with Skyline (V. 3.6, Seattle, WA, USA) originating from University of Washington.

### 4.9. RNA Extraction and qRT-PCR

Total RNA was extracted and isolated from the liver samples by utilizing RNA isolation Kits (QIAGEN, Hilden, Germany), and then qRT-PCR was arranged using a FastStart Universal SYBR Green Master (Roche, Basel, Switzerland) followed by the complementary DNA (cDNA) produced with a PrimeScript RT reagent Kit (TaKaRa, Kusatsu, Japan). We set glyceraldehyde-3-phosphate dehydrogenase (GAPDH) or β-actin as internal references and four biological replicates were conducted for each group. The primer sequences are listed in Table S3. All data were normalized to internal references and analyzed by adopting the 2^−^^ΔΔ^^Ct^ method.

### 4.10. Statistical Analysis

For evaluating two different groups, the Student’s *t*-test was employed, and the data were shown as mean ± SEM (standard error of the mean). A *p-*value of less than 0.05, 0.01 or 0.001 was set as statistically significant.

## 5. Conclusions

In conclusion, by performing proteomic analyses of both the serum and liver of an animal model with NAFLD and diabetes based on *db*/*db* mice, we detected a series of DEPs and analyzed the characteristics of these proteins. In addition, commonly regulated DEPs were selected to confirm their potential as biomarkers for NAFLD. These data provide potential candidate biomarkers for NAFLD, especially for NAFLD with metabolic disorders based on animal models. Furthermore, this work could pave the way for future preclinical therapeutic targets for NAFLD with metabolic dysfunctions.

## Figures and Tables

**Table 1:** The list of clustered serum DEPs identified by MCODE.

MCODE Cluster-ID	Gene Symbol	MCODE Score	Biological Functions of These Genes
Cluster 1	*Psmb3*	7.50	20S proteasome; Proteasome; Cross-presentation of soluble exogenous antigens (endosomes)
	*Psmb2*		
	*Psma7*		
	*Psma6*		
	*Psma5*		
	*Psma4*		
	*Psma1*		
	*Psmb7*		
	*Psmb6*		
	*Psmb5*		
	*Psmb4*		
	*Psmb10*		
	*Psmb1*		
	*Psma3*		
	*Psma2*		
	*Psmb8*		
Cluster 2	*Vgf*	5.06	Complement and coagulation cascades; Regulation of insulin-like growth factor (IGF) transport and uptake by insulin-like growth factor binding proteins (IGFBPs); Post-translational protein phosphorylation
	*Fgb*		
	*Fermt3*		
	*Fgg*		
	*Serpina1e*		
	*Serpina1a*		
	*Serpinf2*		
	*Orm3*		
	*Itih2*		
	*Igfbp3*		
	*Igf1*		
	*Cp*		
	*C4b*		
	*Serping1*		
	*Apoe*		
	*Cfd*		
Cluster 3	*Agxt2*	1.17	Small molecule catabolic process; Neutrophil degranulation; Monocarboxylic acid metabolic process
	*Vnn1*		
	*Sord*		
	*Lamp2*		
	*Lamp1*		
	*Fah*		
Cluster 4	*Rap1b*	2.00	Integrin-mediated cell adhesion; Focal adhesion; Rap1 signaling pathway; Cell adhesion mediated by integrin
	*Itgb3*		
	*Itgb2*		
	*Itgam*		
	*Itga2b*		
Cluster 5	*C8a*	2.00	Terminal pathway of complement; Complement activation, alternative pathway cytolysis
	*C8b*		
	*C8g*		
	*Hc*		
	*C9*		
Cluster 6	*Apom*	1.50	Plasma lipoprotein particle remodeling; Protein–lipid complex remodeling; Protein-containing complex remodeling
	*Pltp*		
	*Apoh*		
	*Apoa4*		
Cluster 7	*Krt12*	1.50	Epidermis development; Formation of the cornified envelope; Keratinization
	*Krt76*		
	*Krt2*		
	*Krt10*		
Cluster 8	*Aldob*	1.50	Carbon metabolism; Hexose metabolic process; Monosaccharide metabolic process
	*H6pd*		
	*Pgam2*		
	*Eno1*		
Cluster 9	*Dstn*	1.00	Actin cytoskeleton organization; Actin-filament-based process
	*Pfn1*		
	*Cap1*		

**Table 2:** DEPs generated by mice serum global proteomics validated by PRM.

Protein Accession	Protein Description	Gene Name	Proteomics (Fold Change)		PRM Validation (Fold Change)	
			srm_db/srm_*bks* Ratio	srm_db/srm_*bks p* Value	srm_db/srm_*bks* Ratio	srm_db/srm *bks p* Value
Q9R1P0	Proteasome subunit alpha type-4 OS = Mus musculus OX = 10,090 GN = Psma4 PE = 1 SV = 1	*Psma4*	1.78	***	2.82	*
O09061	Proteasome subunit beta type-1 OS = Mus musculus OX = 10,090 GN = Psmb1 PE = 1 SV = 1	*Psmb1*	1.86	***	2.83	**
Q9R1P4	Proteasome subunit alpha type-1 OS = Mus musculus OX = 10,090 GN = Psma1 PE = 1 SV = 1	*Psma1*	1.95	***	2.38	*
Q9Z2U0	Proteasome subunit alpha type-7 OS = Mus musculus OX = 10,090 GN = Psma7 PE = 1 SV = 1	*Psma7*	2.04	***	2.29	*
Q91Y97	Fructose-bisphosphate aldolase B OS = Mus musculus OX = 10,090 GN = Aldob PE = 1 SV = 3	*Aldob*	2.93	***	8.08	*
Q00898	Alpha-1-antitrypsin 1–5 OS = Mus musculus OX = 10,090 GN = Serpina1e PE = 1 SV = 1	*Serpina1e*	0.14	***	0.01	**
Q60692	Proteasome subunit beta type-6 OS = Mus musculus OX = 10,090 GN = Psmb6 PE = 1 SV = 3	*Psmb6*	2.01	***	2.99	***
Q9QUM9	Proteasome subunit alpha type-6 OS = Mus musculus OX = 10,090 GN = Psma6 PE = 1 SV = 1	*Psma6*	2.03	***	3.04	**
Q9R1P1	Proteasome subunit beta type-3 OS = Mus musculus OX = 10,090 GN = Psmb3 PE = 1 SV = 1	*Psmb3*	2.12	***	3.43	*
P07758	Alpha-1-antitrypsin 1–1 OS = Mus musculus OX = 10,090 GN = Serpina1a PE = 1 SV = 4	*Serpina1a*	0.47	*	0.46	*

Note: * *p* < 0.05; ** *p* < 0.01; *** *p* < 0.001.

**Table 3:** The list of clustered hepatic DEPs identified by MCODE.

MCODE Cluster-ID	Gene Symbol	MCODE Score	Biological Functions of These Genes
Cluster 1	*Hspa13*	4.33	Protein folding; ER-localized multiprotein complex, in absence of Ig heavy chains ER-localized multiprotein complex, Ig heavy chain-associated;
	*Eif4b*		
	*Dnajc25*		
	*Rps27l*		
	*Nudt7*		
	*Nop56*		
	*Rpl11*		
	*Rsl1d1*		
	*Sdf2l1*		
	*Dnaja4*		
	*Rps28*		
	*Sec61a1*		
	*Pnrc2*		
	*Rps7*		
	*Rps3a1*		
	*Rbm3*		
	*Ppib*		
	*Sqstm1*		
	*Hsp90aa1*		
	*Hspa1b*		
	*Hsph1*		
	*Hspa5*		
	*Pdia3*		
	*Fbl*		
	*Pdia4*		
	*Hyou1*		
Cluster 2	*Cyp2c23*	6.62	Retinol metabolism; Chemical carcinogenesis—DNA adducts; Xenobiotic metabolic process
	*Cyp2c70*		
	*Gstt3*		
	*Gstk1*		
	*Ugt2a3*		
	*Cyp2u1*		
	*Mgst3*		
	*Erg28*		
	*Cyp3a25*		
	*Aldh1a7*		
	*Hsd3b5*		
	*Gstp1*		
	*Gsta2*		
	*Gsta1*		
	*Cyp4a14*		
	*Cyp4a10*		
	*Cyp2b9*		
	*Cyp2b10*		
	*Cyp26a1*		
	*Cyp1a2*		
Cluster 3	*Dnajc3*	4.50	Post-translational protein phosphorylation; Regulation of insulin-like growth factor (IGF) transport and uptake by insulin-like growth factor binding proteins (IGFBPs); Plasma lipoprotein remodeling
	*Pdia6*		
	*Apoa5*		
	*Hsp90b1*		
	*Serpina1e*		
	*Serpina1b*		
	*Calu*		
	*Apoa2*		
	*Alb*		
Cluster 4	*Aldob*	1.90	Glycolysis/gluconeogenesis; Glucose metabolism; Gluconeogenesis
	*H1f5*		
	*Maoa*		
	*H2ax*		
	*H1f3*		
	*Fbp1*		
	*Eno3*		
	*Bpgm*		
	*Aldoc*		
Cluster 5	*Ttll3*	1.25	GnRH secretion; Bacterial invasion of epithelial cells; RHO GTPase effectors
	*Arpc1a*		
	*Trpc4*		
	*Prkca*		
	*Itpr2*		
	*Cdh1*		
	*Casp3*		
	*Arpc1b*		
Cluster 6	*Abcb8*	1.43	Supramolecular fiber organization; Actin cytoskeleton organization; Actin filament-based process
	*Crip2*		
	*Lima1*		
	*Abcb10*		
	*Ppl*		
	*Gas2*		
Cluster 7	*Acss3*	2.50	Valine, leucine and isoleucine degradation; Propanoate metabolism; Carboxylic acid catabolic process
	*Abat*		
	*Aldh1b1*		
	*Hadh*		
	*Acadm*		
Cluster 8	*Txndc5*	2.00	Neutrophil degranulation
	*Dpp7*		
	*Orm2*		
	*Fabp5*		
Cluster 9	*Spcs2*	1.80	Signal peptide processing; Synthesis, secretion, and deacylation of ghrelin; Protein export
	*Sec11c*		
	*Sec11a*		
	*Alpl*		
Cluster 10	*Rpn1*	1.50	Protein N-linked glycosylation; Macromolecule glycosylation; Protein glycosylation
	*Ostc*		
	*Stt3a*		
Cluster 11	*Acot4*	1.50	Peroxisomal protein import; Protein localization; Fatty acid metabolism
	*Acot3*		
	*Acot1*		
Cluster 12	*Akr1d1*	1.50	Steroid hormone biosynthesis; Androgen metabolic process; Steroid catabolic process
	*Hsd17b6*		
	*Hsd17b2*		
Cluster 13	*Necap1*	1.50	Clathrin-mediated endocytosis; Cargo recognition for clathrin-mediated endocytosis; Membrane Trafficking
	*Egfr*		
	*Cttn*		
Cluster 14	*C8b*	1.50	Terminal pathway of complement Complement activation, alternative pathway; Cytolysis
	*C8g*		
	*Hc*		

**Table 4:** DEPs generated by mice liver global proteomics validated by PRM.

Protein Accession	Protein Description	Gene Name	Proteomics (Fold Change)		PRM Validation (Fold Change)	
			lvr_db/lvr_*bks* Ratio	lvr_db/lvr_*bks p* Value	lvr_db/lvr_*bks* Ratio	lvr_db/lvr_*bks p* Value
Q00898	Alpha-1-antitrypsin 1–5 OS = Mus musculus OX = 10,090 GN = Serpina1e PE = 1 SV = 1	*Serpina1e*	0.19	***	0.10	***
Q9ESP1	Stromal cell-derived factor 2-like protein 1 OS = Mus musculus OX = 10,090 GN = Sdf2l1 PE = 1 SV = 2	*Sdf2l1*	0.47	***	0.34	**
P58044	Isopentenyl-diphosphate Delta-isomerase 1 OS = Mus musculus OX = 10,090 GN = Idi1 PE = 1 SV = 1	*Idi1*	0.43	***	0.25	**
Q61694	NADPH-dependent 3-keto-steroid reductase Hsd3b5 OS = Mus musculus OX = 10,090 GN = Hsd3b5 PE = 1 SV = 4	*Hsd3b5*	0.17	***	0.01	*
Q9R092	17-beta-hydroxysteroid dehydrogenase type 6 OS = Mus musculus OX = 10,090 GN = Hsd17b6 PE = 1 SV = 1	*Hsd17b6*	0.41	***	0.19	***
P51658	Estradiol 17-beta-dehydrogenase 2 OS = Mus musculus OX = 10,090 GN = Hsd17b2 PE = 1 SV = 2	*Hsd17b2*	0.41	***	0.18	***
Q99L20	Glutathione S-transferase theta-3 OS = Mus musculus OX = 10,090 GN = Gstt3 PE = 1 SV = 1	*Gstt3*	2.26	***	6.01	***
Q920E5	Farnesyl pyrophosphate synthase OS = Mus musculus OX = 10,090 GN = Fdps PE = 1 SV = 1	*Fdps*	0.48	*	0.27	***
Q91W64	Cytochrome P450 2C70 OS = Mus musculus OX = 10,090 GN = Cyp2c70 PE = 1 SV = 2	*Cyp2c70*	0.34	***	0.10	***
P12790	Cytochrome P450 2B9 OS = Mus musculus OX = 10,090 GN = Cyp2b9 PE = 1 SV = 2	*Cyp2b9*	3.53	***	33.56	***
Q60598	Src substrate cortactin OS = Mus musculus OX = 10,090 GN = Cttn PE = 1 SV = 2	*Cttn*	1.54	**	2.04	***
Q14DH7	Acyl-CoA synthetase short-chain family member 3, mitochondrial OS = Mus musculus OX = 10,090 GN = Acss3 PE = 1 SV = 2	*Acss3*	2.79	***	6.49	***
Q8BWN8	Peroxisomal succinyl-coenzyme A thioesterase OS = Mus musculus OX = 10,090 GN = Acot4 PE = 1 SV = 1	*Acot4*	1.79	***	2.81	***
Q9QYR7	Acyl-coenzyme A thioesterase 3 OS = Mus musculus OX = 10,090 GN = Acot3 PE = 1 SV = 1	*Acot3*	2.70	***	6.99	***
O55137	Acyl-coenzyme A thioesterase 1 OS = Mus musculus OX = 10,090 GN = Acot1 PE = 1 SV = 1	*Acot1*	2.42	***	4.19	***

Note: * *p* < 0.05; ** *p* < 0.01; *** *p* < 0.001.

## Data Availability

Proteomic data is stored in the ProteomeXchange Consortium through the Proteomic Identification (PRIDE) partner repository with accession numbers: PXD027938 for mouse liver and PXD027937 for mouse serum (http://www.ebi.ac.uk/pride, access on 16 August 2021).

## Supplementary Materials

The following supporting information can be downloaded at: https://www.mdpi.com/article/10.3390/ijms23179687/s1.

## References

[1] Liu C., Zhou B., Meng M., Zhao W., Wang D., Yuan Y., Zheng Y., Qiu J., Li Y., Li G. (2021). FOXA3 induction under endoplasmic reticulum stress contributes to non-alcoholic fatty liver disease. J. Hepatol..

[2] Weng J.P. (2020). The definition and classification of metabolic liver disease. Zhonghua Yi Xue Za Zhi.

[3] Eslam M., Sanyal A.J., George J., on behalf of theInternational Consensus Panel (2020). MAFLD: A Consensus-Driven Proposed Nomenclature for Metabolic Associated Fatty Liver Disease. Gastroenterology.

[4] Eslam M., Newsome P.N., Sarin S.K., Anstee Q.M., Targher G., Romero-Gomez M., Zelber-Sagi S., Wong V.W.-S., Dufour J.-F., Schattenberg J.M. (2020). A new definition for metabolic dysfunction-associated fatty liver disease: An international expert consensus statement. J. Hepatol..

[5] Friedman S.L., Neuschwander-Tetri B.A., Rinella M., Sanyal A.J. (2018). Mechanisms of NAFLD development and therapeutic strategies. Nat. Med..

[6] Wong V.W.-S., Adams L.A., De Lédinghen V., Wong G.L.-H., Sookoian S. (2018). Noninvasive biomarkers in NAFLD and NASH—Current progress and future promise. Nat. Rev. Gastroenterol. Hepatol..

[7] Piazzolla V.A., Mangia A. (2020). Noninvasive Diagnosis of Nafld and Nash. Cells.

[8] Di Mauro S., Scamporrino A., Filippello A., Di Pino A., Scicali R., Malaguarnera R., Purrello F., Piro S. (2021). Clinical and Molecular Biomarkers for Diagnosis and Staging of Nafld. Int. J. Mol. Sci..

[9] Niu L., E Geyer P., Albrechtsen N.J.W., Gluud L.L., Santos A., Doll S., Treit P.V., Holst J.J., Knop F.K., Vilsbøll T. (2019). Plasma proteome profiling discovers novel proteins associated with non-alcoholic fatty liver disease. Mol. Syst. Biol..

[10] Zhu W., Zhang Y., Ren C.-H., Cheng X., Chen J.-H., Ge Z.-Y., Sun Z.-P., Zhuo X., Sun F.-F., Chen Y.-L. (2020). Identification of proteomic markers for ram spermatozoa motility using a tandem mass tag (TMT) approach. J. Proteom..

[11] Zhu Y., Aebersold R., Mann M., Guo T. (2021). SnapShot: Clinical proteomics. Cell.

[12] Thompson A., Schäfer J., Kuhn K., Kienle S., Schwarz J., Schmidt G., Neumann T., Hamon C. (2003). Tandem mass tags: A novel quantification strategy for comparative analysis of complex protein mixtures by MS/MS. Anal. Chem..

[13] Canessa E.H., Goswami M.V., Alayi T.D., Hoffman E.P., Hathout Y. (2020). Absolute quantification of dystrophin protein in human muscle biopsies using parallel reaction monitoring (PRM). Biol. Mass Spectrom..

[14] Hu Y., Ye S., Li Q., Yin T., Wu J., He J. (2020). Quantitative Proteomics Analysis Indicates that Upregulation of lncRNA HULC Promotes Pathogenesis of Glioblastoma Cells. OncoTargets Ther..

[15] Liu D., Pan Y., Li K., Li D., Li P., Gao Z. (2020). Proteomics Reveals the Mechanism Underlying the Inhibition of *Phytophthora sojae* by Propyl Gallate. J. Agric. Food Chem..

[16] Yuan T., Cai M., Sheng Y., Ding X., Shen T., Li W., Huang H., Liang B., Zhang X., Zhu Q. (2021). Differentially expressed proteins identified by TMT proteomics analysis in children with verrucous epidermal naevi. J. Eur. Acad. Dermatol. Venereol..

[17] Sahai A., Malladi P., Pan X., Paul R., Melin-Aldana H., Green R.M., Whitington P.F. (2004). Obese and diabetic *db*/*db* mice develop marked liver fibrosis in a model of nonalcoholic steatohepatitis: Role of short-form leptin receptors and osteopontin. Am. J. Physiol. Liver Physiol..

[18] Moriles E.K., Azer S.A. (2022). Alanine Amino Transferase. StatPearls.

[19] Liu H.-W., Kao H.-H., Wu C.-H. (2019). Exercise training upregulates SIRT1 to attenuate inflammation and metabolic dysfunction in kidney and liver of diabetic db/db mice. Nutr. Metab..

[20] Neuman M.G., Cohen L.B., Nanau R.M. (2014). Biomarkers in nonalcoholic fatty liver disease. Can. J. Gastroenterol. Hepatol..

[21] Cao Y., Szabolcs A., Dutta S.K., Yaqoob U., Jagavelu K., Wang L., Leof E.B., Urrutia R.A., Shah V.H., Mukhopadhyay D. (2010). Neuropilin-1 Mediates Divergent R-Smad Signaling and the Myofibroblast Phenotype. J. Biol. Chem..

[22] Arab J.P., Cabrera D., Sehrawat T., Jalan-Sakrikar N., Verma V.K., Simonetto D., Cao S., Yaqoob U., Leon J., Freire M. (2020). Hepatic stellate cell activation promotes alcohol-induced steatohepatitis through Igfbp3 and SerpinA12. J. Hepatol..

[23] Elpek G. (2015). Neuropilins and liver. World J. Gastroenterol..

[24] Bondeva T., Rüster C., Franke S., Hammerschmid E., Klagsbrun M., Cohen C.D., Wolf G. (2009). Advanced glycation end-products suppress neuropilin-1 expression in podocytes. Kidney Int..

[25] Bondeva T., Wolf G. (2015). Role of Neuropilin-1 in Diabetic Nephropathy. J. Clin. Med..

[26] Loeffler I., Rüster C., Franke S., Liebisch M., Wolf G. (2013). Erythropoietin ameliorates podocyte injury in advanced diabetic nephropathy in the db/db mouse. Am. J. Physiol. Ren. Physiol..

[27] Schiekofer S., Galasso G., Sato K., Kraus B.J., Walsh K. (2005). Impaired Revascularization in a Mouse Model of Type 2 Diabetes Is Associated with Dysregulation of a Complex Angiogenic-Regulatory Network. Arter. Thromb. Vasc. Biol..

[28] Zhou Y., Rui L. (2010). Major Urinary Protein Regulation of Chemical Communication and Nutrient Metabolism. Vitam. Horm..

[29] Hayakawa J.I., Nikaido H., Koizumi T. (1983). Components of major urinary proteins (MUP’s) in the mouse: Sex, strain, and subspecies differences. J. Hered..

[30] Shahan K., Denaro M., Gilmartin M., Shi Y., Derman E. (1987). Expression of six mouse major urinary protein genes in the mammary, parotid, sublingual, submaxillary, and lachrymal glands and in the liver. Mol. Cell. Biol..

[31] Shaw P.H., Held W.A., Hastie N.D. (1983). The gene family for major urinary proteins: Expression in several secretory tissues of the mouse. Cell.

[32] Stopková R., Stopka P., Janotová K., Jedelský P.L. (2007). Species-specific Expression of Major Urinary Proteins in the House Mice (Mus musculus musculus and Mus musculus domesticus). J. Chem. Ecol..

[33] Hui X., Zhu W., Wang Y., Lam K.S.L., Zhang J., Wu D., Kraegen E.W., Li Y., Xu A. (2009). Major Urinary Protein-1 Increases Energy Expenditure and Improves Glucose Intolerance through Enhancing Mitochondrial Function in Skeletal Muscle of Diabetic Mice. J. Biol. Chem..

[34] Zhou Y., Jiang L., Rui L. (2009). Identification of MUP1 as a Regulator for Glucose and Lipid Metabolism in Mice. J. Biol. Chem..

[35] Chen C.-C., Lee T.-Y., Kwok C.-F., Hsu Y.-P., Shih K.-C., Lin Y.-J., Ho L.-T. (2015). Major urinary protein 1 interacts with cannabinoid receptor type 1 in fatty acid-induced hepatic insulin resistance in a mouse hepatocyte model. Biochem. Biophys. Res. Commun..

[36] Cho Y.-H., Kim D., Choi I., Bae K. (2011). Identification of transcriptional regulatory elements required for the Mup2 expression in circadian clock mutant mice. Biochem. Biophys. Res. Commun..

[37] Schoeller E.L., Tonsfeldt K.J., Sinkovich M., Shi R., Mellon P.L. (2021). Growth Hormone Pulses and Liver Gene Expression Are Differentially Regulated by the Circadian Clock Gene *Bmal1*. Endocrinology.

[38] Yang H., Zhang W., Lu S., Lu G., Zhang H., Zhuang Y., Wang Y., Dong M., Zhang Y., Zhou X. (2016). Mup-knockout mice generated through CRISPR/Cas9-mediated deletion for use in urinary protein analysis. Acta Biochim. Et Biophys. Sin..

[39] Janciauskiene S., Wrenger S., Immenschuh S., Olejnicka B., Greulich T., Welte T., Chorostowska-Wynimko J. (2018). The Multifaceted Effects of Alpha1-Antitrypsin on Neutrophil Functions. Front. Pharmacol..

[40] Janciauskiene S.M., Bals R., Koczulla R., Vogelmeier C., Köhnlein T., Welte T. (2011). The discovery of α1-antitrypsin and its role in health and disease. Respir. Med..

[41] Fromme M., Schneider C.V., Trautwein C., Brunetti-Pierri N., Strnad P. (2021). Alpha-1 antitrypsin deficiency: A re-surfacing adult liver disorder. J. Hepatol..

[42] Martinez-Huenchullan S.F., Shipsey I., Hatchwell L., Min D., Twigg S.M., Larance M. (2021). Blockade of High-Fat Diet Proteomic Phenotypes Using Exercise as Prevention or Treatment. Mol. Cell. Proteom..

[43] Grander C., Schaefer B., Schwärzler J., Grabherr F., de Graaf D.M., Enrich B., Oberhuber G., Mayr L., Sangineto M., Jaschke N. (2020). Alpha-1 antitrypsin governs alcohol-related liver disease in mice and humans. Gut.

[44] Kalas M.A., Chavez L., Leon M., Taweesedt P.T., Surani S. (2021). Abnormal liver enzymes: A review for clinicians. World J. Hepatol..

[45] Arakaki T.L., Pezza J.A., Cronin M.A., Hopkins C.E., Zimmer D.B., Tolan D.R., Allen K.N. (2004). Structure of human brain fructose 1,6-(bis)phosphate aldolase: Linking isozyme structure with function. Protein Sci..

[46] Nesteruk M., Hennig E., Mikula M., Karczmarski J., Dzwonek A., Goryca K., Rubel T., Paziewska A., Woszczyński M., Ledwon J. (2014). Mitochondrial-related proteomic changes during obesity and fasting in mice are greater in the liver than skeletal muscles. Funct. Integr. Genom..

[47] James C.L., Rellos P., Ali M., Heeley A.F., Cox T.M. (1996). Neonatal screening for hereditary fructose intolerance: Frequency of the most common mutant aldolase B allele (A149P) in the British population. J. Med Genet..

[48] Gruchota J., Pronicka E., Korniszewski L., Stolarski B., Pollak A., Rogaszewska M., Płoski R. (2006). Aldolase B mutations and prevalence of hereditary fructose intolerance in a Polish population. Mol. Genet. Metab..

[49] Coffee E.M., Yerkes L., Ewen E.P., Zee T., Tolan D.R. (2010). Increased prevalence of mutant null alleles that cause hereditary fructose intolerance in the American population. J. Inherit. Metab. Dis..

[50] Oppelt S.A., Sennott E.M., Tolan D.R. (2015). Aldolase-B knockout in mice phenocopies hereditary fructose intolerance in humans. Mol. Genet. Metab..

[51] Lanaspa M.A., Andres-Hernando A., Orlicky D.J., Cicerchi C., Jang C., Li N., Milagres T., Kuwabara M., Wempe M.F., Rabinowitz J.D. (2018). Ketohexokinase C blockade ameliorates fructose-induced metabolic dysfunction in fructose-sensitive mice. J. Clin. Investig..

[52] Murphy S., Yang H., Moylan C.A., Pang H., Dellinger A., Abdelmalek M., Garrett M.E., Ashley-Koch A., Suzuki A., Tillmann H.L. (2013). Relationship Between Methylome and Transcriptome in Patients with Nonalcoholic Fatty Liver Disease. Gastroenterology.

[53] Alonso C., Fernández-Ramos D., Varela-Rey M., Martínez-Arranz I., Navasa N., Van Liempd S.M., Lavín Trueba J.L., Mayo R., Ilisso C.P., de Juan V.G. (2017). Metabolomic Identification of Subtypes of Nonalcoholic Steatohepatitis. Gastroenterology.

[54] Moylan C.A., Pang H., Dellinger A., Suzuki A., Garrett M.E., Guy C.D., Murphy S.K., Ashley-Koch A.E., Choi S.S., Michelotti G. (2014). Hepatic gene expression profiles differentiate presymptomatic patients with mild versus severe nonalcoholic fatty liver disease. Hepatology.

[55] Turecky L., Kupcova V., Durfinova M., Uhlikova E. (2021). Serum butyrylcholinesterase activities in patients with non-alcoholic fatty liver disease. Comparison with liver proteosynthetic function and liver fibrosis. Bratisl. Med J..

[56] Hardwick R.N., Fisher C.D., Canet M.J., Scheffer G.L., Cherrington N.J. (2011). Variations in ATP-Binding Cassette Transporter Regulation during the Progression of Human Nonalcoholic Fatty Liver Disease. Drug Metab. Dispos..

[57] Chen Y.-M., Liu Y., Zhou R.-F., Chen X.-L., Wang C., Tan X.-Y., Wang L.-J., Zheng R.-D., Zhang H.-W., Ling W.-H. (2016). Associations of gut-flora-dependent metabolite trimethylamine-N-oxide, betaine and choline with non-alcoholic fatty liver disease in adults. Sci. Rep..

[58] Huang T., Yu L., Pan H., Ma Z., Wu T., Zhang L., Liu K., Qi Q., Miao W., Song Z. (2021). Integrated Transcriptomic and Translatomic Inquiry of the Role of Betaine on Lipid Metabolic Dysregulation Induced by a High-Fat Diet. Front. Nutr..

[59] Fan C., Hu H., Huang X., Su D., Huang F., Zhuo Z., Tan L., Xu Y., Wang Q., Hou K. (2022). Betaine Supplementation Causes an Increase in Fatty Acid Oxidation and Carbohydrate Metabolism in Livers of Mice Fed a High-Fat Diet: A Proteomic Analysis. Foods.

[60] Sy S.M.-H., Lai P.B.-S., Pang E., Wong N.L.-Y., To K.-F., Johnson P.J., Wong N. (2006). Novel identification of zyxin upregulations in the motile phenotype of hepatocellular carcinoma. Mod. Pathol..

[61] Bergen H.R., Vasmatzis G., Cliby W.A., Johnson K.L., Oberg A.L., Muddiman D.C. (2003). Discovery of ovarian cancer biomarkers in serum using NanoLC electrospray ionization TOF and FT-ICR mass spectrometry. Dis. Markers.

[62] El-Hattab A.W., Dai H., Almannai M., Wang J., Faqeih E.A., Al Asmari A., Saleh M.A.M., Elamin M.A.O., Alfadhel M., Alkuraya F.S. (2017). Molecular and clinical spectra of FBXL4 deficiency. Hum. Mutat..

[63] Shinomiya H. (2012). Plastin Family of Actin-Bundling Proteins: Its Functions in Leukocytes, Neurons, Intestines, and Cancer. Int. J. Cell Biol..

[64] Zhang T., Wang Z., Liu Y., Huo Y., Liu H., Xu C., Mao R., Zhu Y., Liu L., Wei D. (2020). Plastin 1 drives metastasis of colorectal cancer through the IQGAP1/Rac1/ERK pathway. Cancer Sci..

[65] Wang F., Kohan A.B., Lo C.-M., Liu M., Howles P., Tso P. (2015). Apolipoprotein A-IV: A protein intimately involved in metabolism. J. Lipid Res..

[66] Kang M., Kim J., An H., Ko J. (2017). Human leucine zipper protein promotes hepatic steatosis via induction of apolipoprotein A-IV. FASEB J..

[67] Wang P.-W., Hung Y.-C., Wu T.-H., Chen M.-H., Yeh C.-T., Pan T.-L. (2017). Proteome-based identification of apolipoprotein A-IV as an early diagnostic biomarker in liver fibrosis. Oncotarget.

[68] Supuran C.T. (2011). Carbonic anhydrase inhibitors and activators for novel therapeutic applications. Futur. Med. Chem..

[69] Supuran C.T. (2008). Carbonic anhydrases—An overview. Curr. Pharm. Des..

[70] Yuan L., Wang M., Liu T., Lei Y., Miao Q., Li Q., Wang H., Zhang G., Hou Y., Chang X. (2019). Carbonic Anhydrase 1-Mediated Calcification Is Associated With Atherosclerosis, and Methazolamide Alleviates Its Pathogenesis. Front. Pharmacol..

[71] Arakawa T., Kobayashi-Yurugi T., Alguel Y., Iwanari H., Hatae H., Iwata M., Abe Y., Hino T., Ikeda-Suno C., Kuma H. (2015). Crystal structure of the anion exchanger domain of human erythrocyte band 3. Science.

[72] Romero M.F., Chen A.P., Parker M.D., Boron W.F. (2013). The SLC4 family of bicarbonate (HCO_3_^−^) transporters. Mol. Asp. Med..

[73] Remigante A., Spinelli S., Pusch M., Sarikas A., Morabito R., Marino A., Dossena S. (2022). Role of SLC4 and SLC26 solute carriers during oxidative stress. Acta Physiol..

[74] Bhushan B., Michalopoulos G.K. (2020). Role of epidermal growth factor receptor in liver injury and lipid metabolism: Emerging new roles for an old receptor. Chem. Interact..

[75] Collin de l’Hortet A., Zerrad-Saadi A., Prip-Buus C., Fauveau V., Helmy N., Ziol M., Vons C., Billot K., Baud V., Gilgenkrantz H. (2014). GH administration rescues fatty liver regeneration impairment by restoring GH/EGFR pathway deficiency. Endocrinology.

[76] Giraudi P.J., Giuricin M., Bonazza D., de Manzini N., Tiribelli C., Palmisano S., Rosso N. (2021). Modifications of IGF2 and EGFR plasma protein concentrations in NAFLD patients after bariatric surgery. Int. J. Obes..

[77] Giraldez M.D., Carneros D., Garbers C., Rose-John S., Bustos M. (2021). New insights into IL-6 family cytokines in metabolism, hepatology and gastroenterology. Nat. Rev. Gastroenterol. Hepatol..

[78] Zhang C., Liu J., Wang J., Hu W., Feng Z. (2021). The emerging role of leukemia inhibitory factor in cancer and therapy. Pharmacol. Ther..

[79] Yuan Y., Li K., Teng F., Wang W., Zhou B., Zhou X., Lin J., Ye X., Deng Y., Liu W. (2022). Leukemia inhibitory factor protects against liver steatosis in nonalcoholic fatty liver disease patients and obese mice. J. Biol. Chem..

[80] Meng Q., Li X., Xiong X. (2022). Identification of Hub Genes Associated with Non-alcoholic Steatohepatitis Using Integrated Bioinformatics Analysis. Front. Genet..

[81] Stanley T.L., Fourman L.T., Zheng I., McClure C.M., Feldpausch M.N., Torriani M., Corey K.E., Chung R.T., Lee H., Kleiner D.E. (2021). Relationship of IGF-1 and IGF-Binding Proteins to Disease Severity and Glycemia in Nonalcoholic Fatty Liver Disease. J. Clin. Endocrinol. Metab..

[82] Fahlbusch P., Knebel B., Hörbelt T., Barbosa D.M., Nikolic A., Jacob S., Al-Hasani H., Van De Velde F., Van Nieuwenhove Y., Müller-Wieland D. (2020). Physiological Disturbance in Fatty Liver Energy Metabolism Converges on IGFBP2 Abundance and Regulation in Mice and Men. Int. J. Mol. Sci..

[83] Yang J., Zhou W., Wu Y., Xu L., Wang Y., Xu Z., Yang Y. (2020). Circulating IGFBP-2 levels are inversely associated with the incidence of nonalcoholic fatty liver disease: A cohort study. J. Int. Med. Res..

[84] Hou W., Janech M.G., Sobolesky P.M., Bland A.M., Samsuddin S., Alazawi W., Syn W.-K. (2020). Proteomic screening of plasma identifies potential noninvasive biomarkers associated with significant/advanced fibrosis in patients with nonalcoholic fatty liver disease. Biosci. Rep..

[85] Hu W., Wang M., Yin C., Li S., Liu Y., Xiao Y. (2018). Serum complement factor 5a levels are associated with nonalcoholic fatty liver disease in obese children. Acta Paediatr..

[86] Hillebrandt S., Wasmuth H.E., Weiskirchen R., Hellerbrand C., Keppeler H., Werth A., Schirin-Sokhan R., Wilkens G., Geier A., Lorenzen J. (2005). Complement factor 5 is a quantitative trait gene that modifies liver fibrogenesis in mice and humans. Nat. Genet..

[87] Viiklepp K., Nissinen L., Ojalill M., Riihilä P., Kallajoki M., Meri S., Heino J., Kähäri V.-M. (2022). C1r Upregulates Production of Matrix Metalloproteinase-13 and Promotes Invasion of Cutaneous Squamous Cell Carcinoma. J. Investig. Dermatol..

[88] Fukuda S., Sumii M., Masuda Y., Takahashi M., Koike N., Teishima J., Yasumoto H., Itamoto T., Asahara T., Dohi K. (2001). Murine and human SDF2L1 is an endoplasmic reticulum stress-inducible gene and encodes a new member of the Pmt/rt protein family. Biochem. Biophys. Res. Commun..

[89] Nonogaki K., Kaji T. (2020). Whey protein isolate inhibits hepatic FGF21 production, which precedes weight gain, hyperinsulinemia and hyperglycemia in mice fed a high-fat diet. Sci. Rep..

[90] Meunier L., Usherwood Y.-K., Chung K.T., Hendershot L.M. (2002). A Subset of Chaperones and Folding Enzymes Form Multiprotein Complexes in Endoplasmic Reticulum to Bind Nascent Proteins. Mol. Biol. Cell.

[91] Bies C., Blum R., Dudek J., Nastainczyk W., Oberhauser S., Jung M., Zimmermann R. (2004). Characterization of pancreatic ERj3p, a homolog of yeast DnaJ-like protein Scj1p. Biol. Chem..

[92] Tongaonkar P., Selsted M.E. (2009). SDF2L1, a Component of the Endoplasmic Reticulum Chaperone Complex, Differentially Interacts with α-, β-, and θ-Defensin Propeptides. J. Biol. Chem..

[93] Walter P., Ron D. (2011). The Unfolded Protein Response: From Stress Pathway to Homeostatic Regulation. Science.

[94] Sasako T., Ohsugi M., Kubota N., Itoh S., Okazaki Y., Terai A., Kubota T., Yamashita S., Nakatsukasa K., Kamura T. (2019). Hepatic Sdf2l1 controls feeding-induced ER stress and regulates metabolism. Nat. Commun..

[95] Schott A., Ravaud S., Keller S., Radzimanowski J., Viotti C., Hillmer S., Sinning I., Strahl S. (2010). Arabidopsis Stromal-derived Factor2 (SDF2) Is a Crucial Target of the Unfolded Protein Response in the Endoplasmic Reticulum. J. Biol. Chem..

[96] Loveland J.L., Lank D.B., Küpper C. (2021). Gene Expression Modification by an Autosomal Inversion Associated with Three Male Mating Morphs. Front. Genet..

[97] Huang X.F., Luu-The V. (2000). Molecular characterization of a first human 3(α→β)-hydroxysteroid epimerase. J. Biol. Chem..

[98] Huang X.F., Luu-The V. (2001). Gene structure, chromosomal localization and analysis of 3-ketosteroid reductase activity of the human 3(α→β)-hydroxysteroid epimerase. Biochim. Biophys. Acta.

[99] Chan Y.X., Yeap B.B. (2018). Dihydrotestosterone and cancer risk. Curr. Opin. Endocrinol. Diabetes Obes..

[100] Kohl M., Wiese S., Warscheid B. (2011). Cytoscape: Software for Visualization and Analysis of Biological Networks. Comput. Aided Tissue Eng..

[101] Bader G.D., Hogue C.W.V. (2003). An automated method for finding molecular complexes in large protein interaction networks. BMC Bioinform..

[102] Zhou Z., Li Y., Hao H., Wang Y., Zhou Z., Wang Z., Chu X. (2019). Screening Hub Genes as Prognostic Biomarkers of Hepatocellular Carcinoma by Bioinformatics Analysis. Cell Transpl..

[103] Huang D.W., Sherman B.T., Lempicki R.A. (2009). Systematic and integrative analysis of large gene lists using DAVID bioinformatics resources. Nat. Protoc..

[104] Szklarczyk D., Gable A.L., Lyon D., Junge A., Wyder S., Huerta-Cepas J., Simonovic M., Doncheva N.T., Morris J.H., Bork P. (2019). STRING v11: Protein-protein association networks with increased coverage, supporting functional discovery in genome-wide experimental datasets. Nucleic Acids Res..

[105] Li M., Zhang K., Long R., Sun Y., Kang J., Zhang T., Cao S. (2017). iTRAQ-based comparative proteomic analysis reveals tissue-specific and novel early-stage molecular mechanisms of salt stress response in Carex rigescens. Environ. Exp. Bot..

