# Supplementary material for: Comparative Proteomic Analysis of Liver Tissues and Serum in db/db Mice
Source: Int J Mol Sci. 2022 Aug 26;23(17):9687. doi: 10.3390/ijms23179687 (PMC9455973; doi:10.3390/ijms23179687)
Supplement: Supplementary file 1 [file ijms-23-09687-s001.zip › 20220826-Yu Zhang-Supplementary Material-1.pdf]

Comparative proteomic analysis of liver tissues and serum in *db/db* mice

Yu Zhang[1], Xiumei Wu[2], Mengyun Xu[1], Tong Yue[1], Ping Ling[1], Tingyu Fang[1], Sihui Luo[1], Suowen Xu[1], Jianping Weng[1*]

Affiliation

[1] Department of Endocrinology, Institute of Endocrine and Metabolic Diseases, The First Affiliated Hospital of USTC, Division of Life Sciences and Medicine, Clinical Research Hospital of Chinese Academy of Sciences (Hefei), University of Science and Technology of China, Hefei, 230001, China

[2] Department of Endocrinology and Metabolic Disease, The Third Affiliated Hospital of Sun Yat-sen University, Guangzhou 510000, China.

Yu Zhang, ORCID ID： 0000-0002-6669-5496

Xiumei Wu, ORCID ID： 0000-0002-9549-7341

Mengyun Xu, ORCID ID： 0000-0001-6544-0777

Tong Yue, ORCID ID： 0000-0002-7203-6948

Ping Ling, ORCID ID： 0000-0002-9889-5812

Tingyu Fang, ORCID ID： 0000-0002-1399-1320

Sihui Luo, ORCID ID： 0000-0001-8503-0310

Suowen Xu, ORCID ID： 0000-0002-5488-5217

Jianping Weng, ORCID ID： 0000-0002-7889-1697

***Correspondence to:**

Jianping Weng, MD, PhD

Email: wengjp@ustc.edu.cn

Address: 96, Jinzhai Rd, Hefei, China, 230026

Supplementary Materials

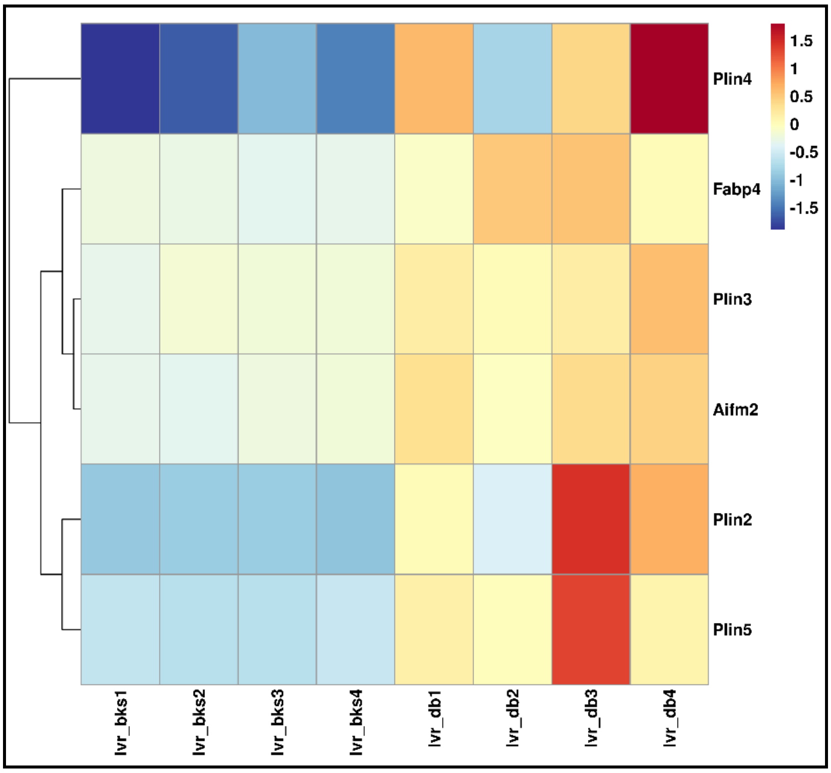

Figure S1. The heatmap of the upregulated lipid droplets related markers in the livers of *db/db* and *bks* mice.

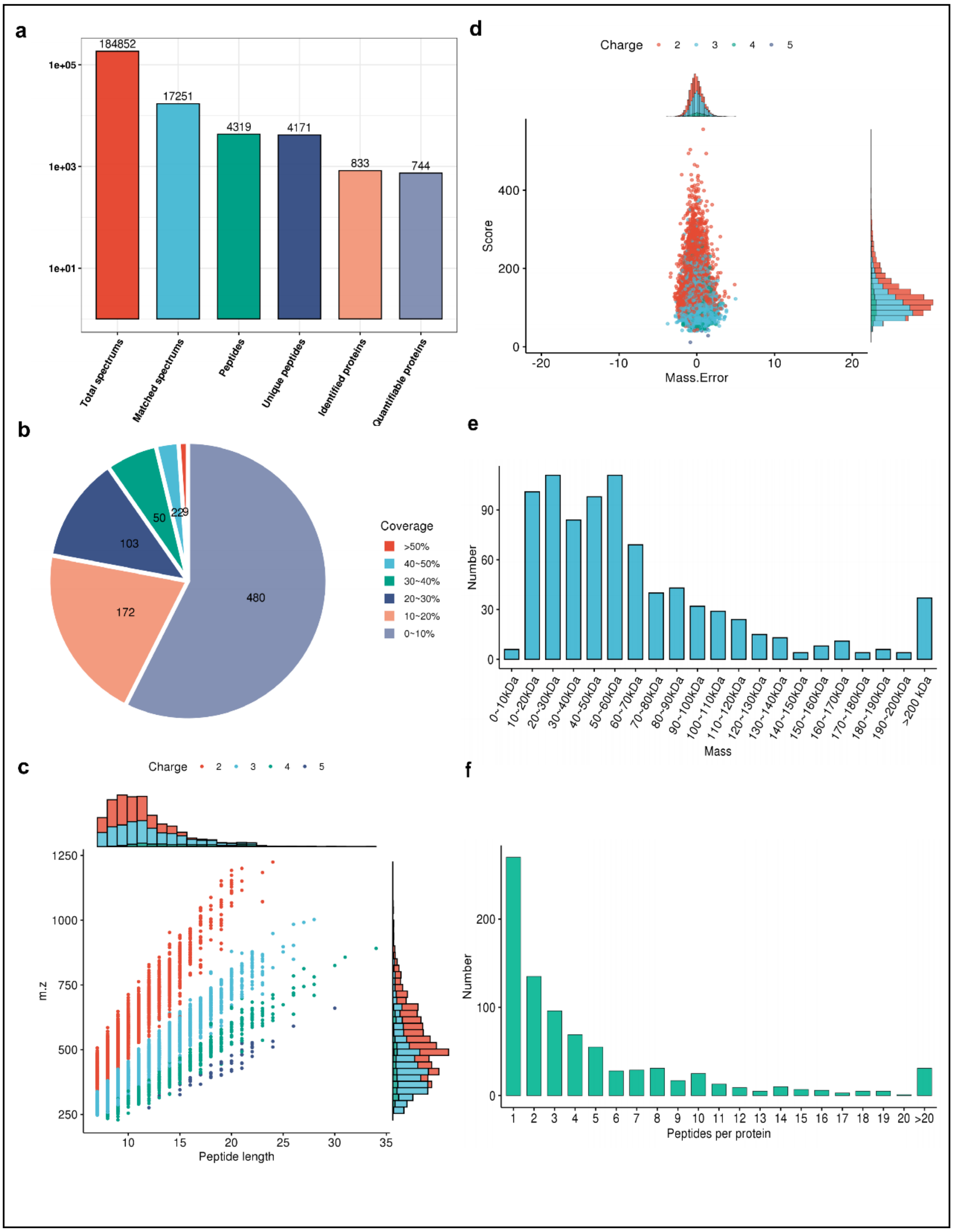

Figure S2. Quantitative protein filtering and data quality control of the serum samples. (a) Results of quantitative proteins filtered from search database; (b) Quality control of protein coverage distribution; (c) Peptide length distribution; (d) Tolerance distribution of parent ion mass; (e) Peptide number distribution; (f) Protein molecular weight distribution.

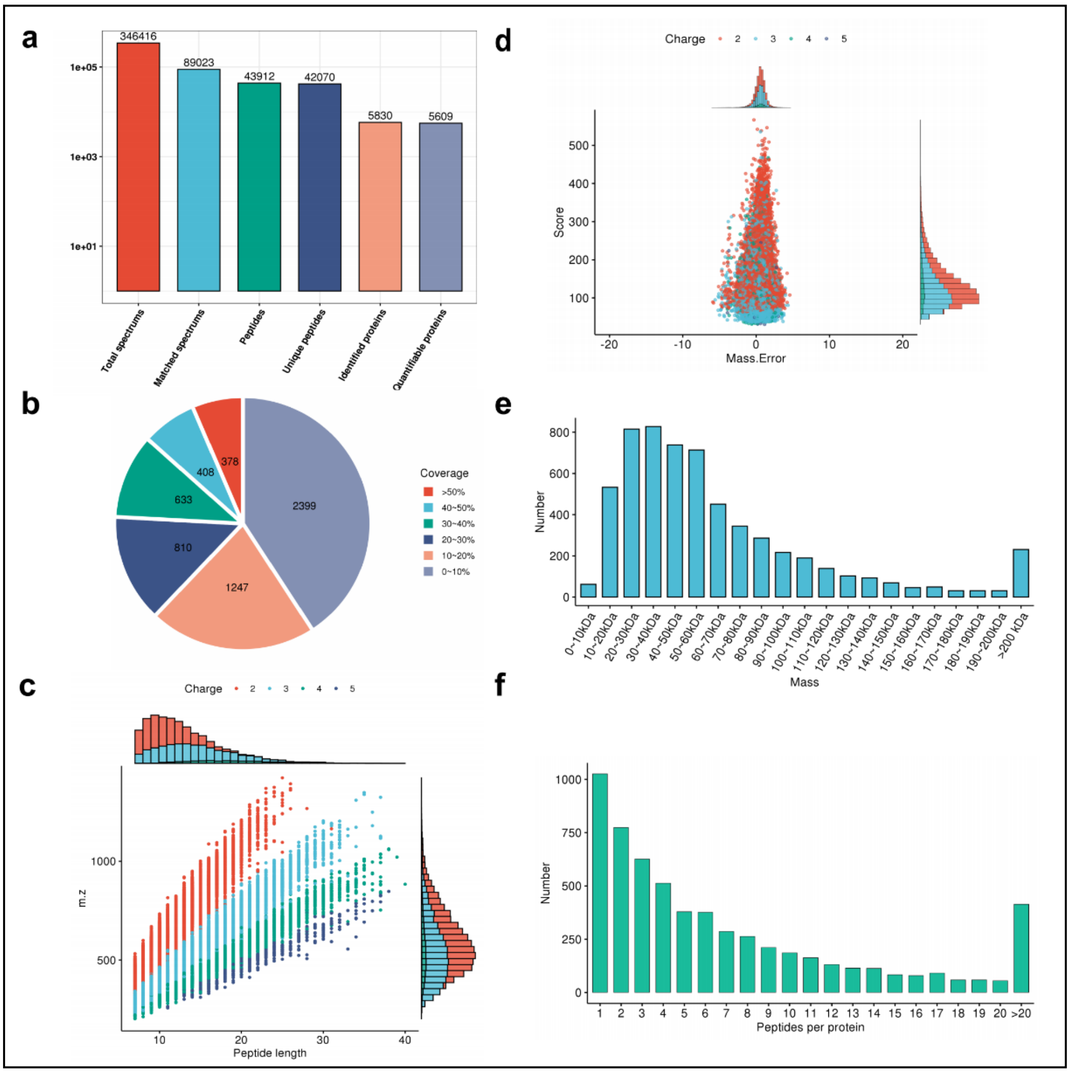

Figure S3. Quantitative protein filtering and data quality control of the liver samples. (a) Results of quantitative proteins filtered from search database; (b) Quality control of protein coverage distribution; (c) Peptide length distribution; (d) Tolerance distribution of parent ion mass; (e) Peptide number distribution; (f) Protein molecular weight distribution.

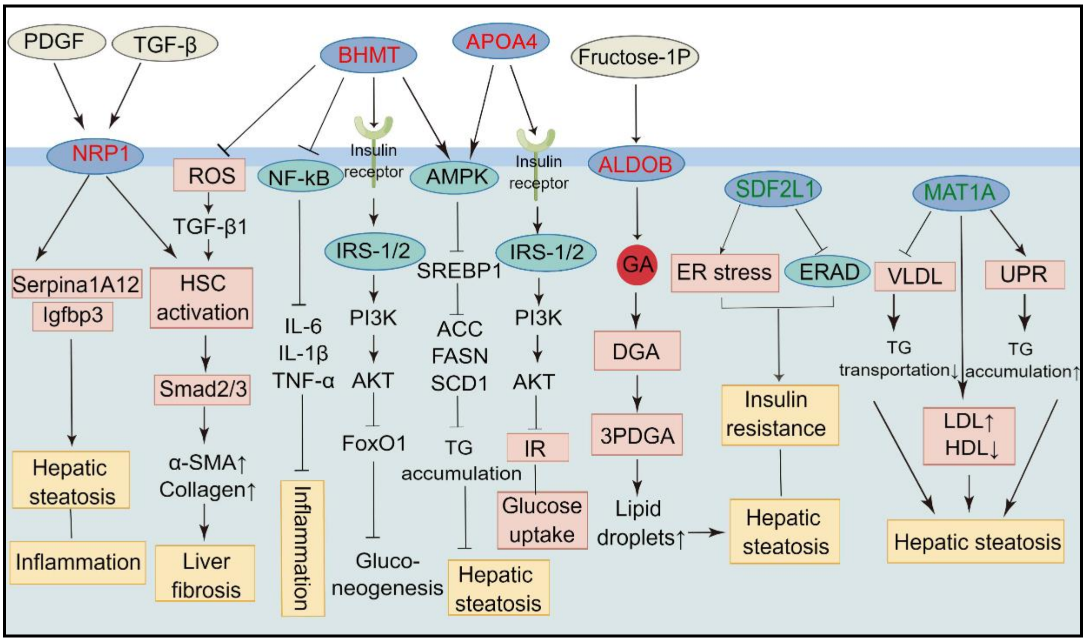

Figure S4. Graphical pathway of how the major DEP markers that are upregulated and downregulated connect to NAFLD. Abbreviations: GA: D-glyceraldehyde. DGA: D-glyceric acid. 3PDGA: 3-phospho-D-glyceric acid. VLDL: Very low-density lipoprotein. UPR: Unfolded protein response. ERAD: Endoplasmic reticulum-associated protein degradation. IR: Insulin resistance. ACC: Acetyl CoA carboxylase. FASN: Fatty acid synthetase. SCD1: Stearoyl-CoA desaturase 1. SREBP1: Sterol regulatory element binding protein 1. IRS-1/2: Insulin receptor substrate-1/2. HSC: Hepatic stellate cells. AMPK: AMP-activated protein kinase. PI3K: Phosphoinositide 3-kinase. AKT: Protein Kinase B**.** Protein name in red represents DEPs were upregulated while in green means downregulated in the pathway involved in NAFLD. The figure is constructed by Figdraw (www.figdraw.com).

Table S1. DEPs related to lipid droplets generated by mice liver global proteomics

Protein accession	Protein description	Gene name	lvr_db/lvr_bks Ratio	lvr_db/lvr_bks P value	Regulated Type
P04117	Fatty acid-binding protein, adipocyte OS=Mus musculus OX=10090 GN=Fabp4 PE=1 SV=3	*Fabp4*	1.484	*	Up
O88492	Perilipin-4 OS=Mus musculus OX=10090 GN=Plin4 PE=1 SV=2	*Plin4*	4.853	*	Up
P43883	Perilipin-2 OS=Mus musculus OX=10090 GN=Plin2 PE=1	*Plin2*	2.861	*	Up

	SV=2				
Q9DBG5	Perilipin-3 OS=Mus musculus OX=10090 GN=Plin3 PE=1 SV=1	*Plin3*	1.381	*	Up
Q8BVZ1	Perilipin-5 OS=Mus musculus OX=10090 GN=Plin5 PE=1 SV=1	*Plin5*	2.233	*	Up
Q8BUE4	Ferroptosis suppressor protein 1 OS=Mus musculus OX=10090 GN=Aifm2 PE=1 SV=1	*Aifm2*	1.472	**	Up

Note: $^{*}P$<0.05; $^{**}P$<0.01; $^{***}P$<0.001.

Table S2. The serum DEPs related to liver damage

**Protein accession**	**Protein description**	**Gene name**	**srm_db/srm_bks Ratio**	**srm_db/srm_bks P value**	**Regulated Type**
P05201	Aspartate aminotransferase, cytoplasmic OS=Mus musculus OX=10090 GN=Got1 PE=1 SV=3	*Got1*	1.967	*	Up
Q8BGT5	Alanine aminotransferase 2 OS=Mus musculus OX=10090 GN=Gpt2 PE=1 SV=1	*Gpt2*	1.417	**	Up
P09242	Alkaline phosphatase, tissue-nonspecific isozyme OS=Mus musculus OX=10090 GN=Alpl PE=1 SV=2	*Alpl*	1.485	**	Up

Note: $^{*}P$<0.05; $^{**}P$<0.01; $^{***}P$<0.001.

Table S3. Sequences of primers

Gene name	Species	Sense sequence	Anti-sense sequence
Nrp1	Mouse	GTGAGCCCTGTGGTCTATTC	GGCTTCTGGTAGCGTAGTTT
Mup3	Mouse	CTATGGCCGAGAACCAGATTT	GCAGCGATTGACATTGGTTAG
Serpina1e	Mouse	ACAGCCTTTGCTATGCTCTC	GATGTCAGCCTCCGATGTTT

Igfbp2	Mouse	GAACATGTTGGGAGGTG GTAG	TGCCGGTGCTGTTCATT
Alpl	Mouse	GCCATGACATCCCAGAA AGA	GCCAGACCAAAGATGG AGTT
Aldob	Mouse	CCAGCCTTGCTATCCAAG AA	GCACCTCTGGCTCAAC AATA
GAPDH	Mouse	AACAGCAACTCCCACTC TTC	CCTGTTGCTGTAGCCGT ATT
β-Actin	Mouse	GAGGTATCCTGACCCTGA AGTA	CACACGCAGCTCATTG TAGA
